# Identifiability of Level-1 Species Networks from Gene Tree Quartets

**DOI:** 10.1007/s11538-024-01339-4

**Published:** 2024-07-25

**Authors:** Elizabeth S. Allman, Hector Baños, Marina Garrote-Lopez, John A. Rhodes

**Affiliations:** 1 Department of Mathematics and Statistics, University of Alaska, Fairbanks, AK USA; 2 Department of Mathematics, California State University San Bernadino, San Bernadino, CA USA; 3 Max Planck Institute for Mathematics in the Sciences, Leipzig, Germany

**Keywords:** Species network, Coalescent model, Concordance factors, Identifiability, 92D15, 92B10, 13P25

## Abstract

When hybridization or other forms of lateral gene transfer have occurred, evolutionary relationships of species are better represented by phylogenetic networks than by trees. While inference of such networks remains challenging, several recently proposed methods are based on quartet concordance factors—the probabilities that a tree relating a gene sampled from the species displays the possible 4-taxon relationships. Building on earlier results, we investigate what level-1 network features are identifiable from concordance factors under the network multispecies coalescent model. We obtain results on both topological features of the network, and numerical parameters, uncovering a number of failures of identifiability related to 3-cycles in the network. Addressing these identifiability issues is essential for designing statistically consistent inference methods.

## Introduction

Statistical inference of phylogenetic networks, showing evolutionary relationships between species when hybridization or other horizontal gene transfer has occurred, poses substantial theoretical and practical problems. With data in the form of many sequenced and aligned genes, standard phylogenetic methods can be used to infer gene trees. However, due to both horizontal inheritance and the population genetic effect of incomplete lineage sorting, these gene trees reflect the species network topology only indirectly. Extracting the network signal with an acceptable computational time, and even determining what aspects of the network can be inferred under the Network Multispecies Coalescent (NMSC) model, is challenging.

Several recently-developed network inference methods utilize summaries of (inferred) gene trees through counts of their displayed quartet trees, that is, empirical quartet *concordance factors* (*CFs*). SNaQ (Solís-Lemus and Ané 2016) uses pseudolikelihood on these *CF*s to pick an optimal network among those of level 1. NANUQ (Allman et al. 2019) also uses quartet counts in the level-1 setting, but avoids pseudolikelihood computations, by conducting hypothesis tests for each quartet, followed by a distance-based approach to avoid searching over networks. (PhyloNet Yu and Nakhleh 2015 similarly uses pseudolikelihood, though with rooted triple counts and without the level-1 restriction).

While these methods strike a balance between thorough statistical analysis and computational effort, a complete exploration of what level-1 network features are identifiable from CFs under the NMSC has yet to be undertaken. First results in this direction (Solís-Lemus and Ané 2016) showed certain semidirected level-1 network topologies were distinguishable from those obtained by dropping a hybrid edge, and that in some cases numerical parameters were identifiable up to a finite number of possibilities, i.e., were locally identifiable. Topological identifiability was later investigated (Baños 2019), establishing that semidirected level-1 network topologies are identifiable up to contraction of 2- and 3-cycles and directions of hybrid edges in 4-cycles, for generic parameters. While these works provide our starting point, we seek to fill in unaddressed gaps. Appendix A gives more detail on how this work complements its predecessors, and discusses the claims and arguments in Solis-Lemus et al. (2020) for work described in Solís-Lemus and Ané (2016).

We rigorously establish what can be identified, and what cannot, from quartet *CF*s under the NMSC. Our concern here is with the theoretical question of identifiability. We thus delineate what *might* be consistently inferred by a method using quartet counts, although particular methods may not be able to do so. Our results also imply parameter identifiability results for data types from which quartet counts can be obtained (e.g., topological gene trees, or metric gene trees), although for such data it is possible that stronger identifiability claims could be established.

Our main results address identifiability of the full semidirected topology of a binary network, including hybrid edge directions (Theorem 19), and the numerical parameters of edge lengths and hybridization (or inheritance) probabilities (Theorem 30). One interesting aspect is that the presence of a 3-cycle can generally be detected, but whether the hybrid node of that cycle can be identified or not depends on the numerical parameters. The subsets of parameters on which this question has a positive or negative answer both have positive measure, and thus neither set can be dismissed as non-generic. This means, in particular, that the direction of gene flow between recently diverged populations may simply be unknowable from CFs in some instances. (We do not, however, suggest CFs be abandoned as a useful data summary, as the limits of identifiability from alternative data summaries has not been similarly investigated.)

The precise statements of these main theorems have exceptions for cycles adjacent to pendant edges. However, these simplify if one has multiple samples per taxon. Then the semidirected network topology is generically identifiable except for the presence of 2-cycles and (sometimes) hybrid nodes in 3-cycles. If the semidirected network topology with 2-cycles removed is known, then all numerical parameters except those relating to 3-cycles and their adjacent edges are generically identifiable.

Underlying our results are analyses of algebraic varieties associated with certain small networks using computational algebra software. These lead to algebraic (polynomial equality) tests of quartet *CF*s for different network substructures. However, 3-cycle identifiability results depend on semialgebraic tests (polynomial inequalities). These tests were motivated by equalities found for related networks, but their construction is not purely computational.

Our identifiability results for numerical parameters are based on explicit rational formulas for parameters in terms of *CF*s, so if a topological network is known or proposed, one could in principle estimate numerical parameters with them. But while some of these formulas are simple, others are quite complicated, and should not be expected to provide good estimates from data. These formulas may, however, give useful initial estimates of parameters that could then be refined through optimization, such as with likelihood methods.

Sections 2 and 3 give definitions and earlier results that we use as our starting point. In Sect. 4 we study topological identifiability of level-1 binary networks from quartet *CF*s, and in Sect. 5 the identifiability of numerical network parameters from the same information. Section 6 discusses implications for data analysis.

Appendix A explains how our results complement earlier work, and Appendix B catalogs the computational results for specific networks that underly our arguments.

## Definitions

### Rooted and Unrooted Phylogenetic Networks

A *topological binary rooted phylogenetic network* $$N^ + $$ is a finite rooted graph, with all edges directed away from the root, whose non-root internal nodes form two classes: *Tree nodes* have indegree 1 and outdegree 2, while *hybrid nodes* have indegree 2 and outdegree 1. *Hybrid edges* and *tree edges* are classified according to their child nodes. Leaves of the network are bijectively labelled by *taxa* in a set *X*. A network is *metric* if in addition each tree edge is assigned a positive length, each hybrid edge a non-negative length and a positive probability $$\gamma $$, such that for every pair of hybrid edges $$e, e'$$ with a common child $$\gamma +\gamma '=1$$. More formal definitions of phylogenetic networks appear in Solís-Lemus and Ané (2016), Baños (2019), Steel (2016).

We often depict these networks with their root at the top, referring to edges and nodes as *above* or *below* one another in the natural way.

As explained in Ané et al. (2024), for gene quartet-based methods of inference a useful form of an unrooted network is more subtle than that for a tree. Substructures above the *least stable ancestor* (LSA) of the taxa Steel (2016) are undetectable by these methods, as is the LSA itself. The *topological unrooted phylogenetic network induced from* $$N^ + $$, is the semidirected network $$N=N^-$$, obtained from $$N^ + $$ by deleting all vertices and nodes above the LSA, undirecting tree edges, and suppressing the LSA. Since our concern in this work is the identifiability of unrooted phylogenetic networks, we will often use *N* rather than the more cumbersome $$N^-$$ to denote them. We refer to *N* as unrooted or semidirected interchangeably. Note that *N* naturally inherits a metric structure if $$N^+$$ has one.

**Fig. 1:**
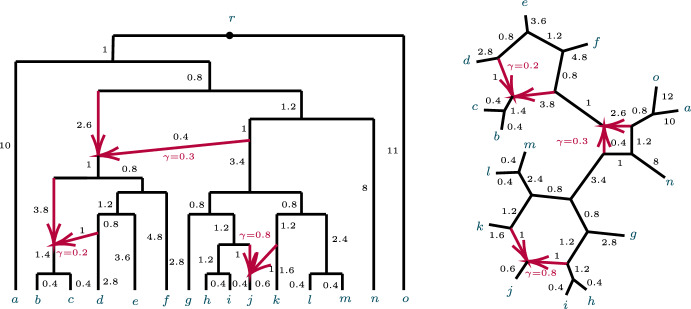
(left) A rooted network $$N^ + $$ on *X* with root $$r = \text {LSA}(X)$$, and (right) the unrooted network $$N^-$$ obtained from $$N^ + $$

Figure 1 shows an example of a network $N^+$ and its semidirected network $N^-$. While in that example all leaves are equidistant from the root of $N^+$, we do not assume ultrametricity generally.

Tree edges can be further partitioned into *cut* and *noncut* edges, according to whether their deletion results in a graph with 2 connected components or not. Note that hybrid edges are never cut edges.

Of particular interest are unrooted networks on four taxa obtained from a larger network by restricting the taxon set. Recall that a binary unrooted topological tree on four taxa *a*, *b*, *c*, *d* is called a *quartet* *ab*|*cd* if deletion of its sole internal edge gives connected components with taxa $\{a, b\}$ and $\{c,\!d\}$. When $n \ge 4$, an *n*-taxon tree *displays* a quartet *ab*|*cd* if the induced unrooted tree on the four taxa is *ab*|*cd*. A formal extension of this concept to quartet networks follows.

#### Definition 1

Let $$N^ + $$ be a rooted network on *X*, and let $$a,b,c,d\in X$$. The *induced quartet network* $$N|_{Q}$$ on $$Q = \{a,b,c,d\}$$ is the unrooted network obtained by

1. Retaining only the nodes and edges of $$N^ + $$ ancestral to at least one of *a*, *b*, *c*, *d*,
2. Suppressing nodes of degree 2, and
3. Unrooting the resulting network.

If $$N^ + $$ is a metric network, its quartet networks naturally are as well. If $$Y \subset X$$ with $$|Y| \ge 5$$, then the *induced subnetwork* $$N|_Y$$ is defined similarly.

**Fig. 2:**
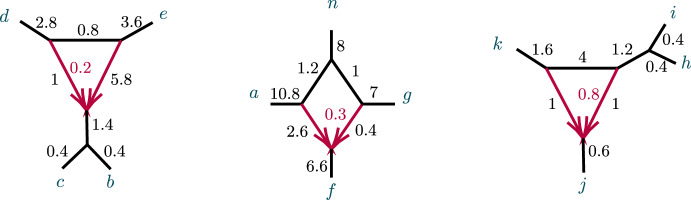
Several semidirected quartet networks induced from the network in Fig. 1

An analogous definition for induced quartet networks of *N* is given in Baños (2019), which also shows that the quartet networks induced from $$N^ + $$ and *N* are isomorphic. Figure 2 shows some metric quartet networks induced from the networks of Fig. 1.

### Level-1 Networks

We restrict our study to the family of level-1 phylogenetic networks. These have been the focus of many works (Solís-Lemus and Ané 2016; Allman et al. 2019; Baños 2019; Huber et al. 2017; Huson et al. 2010; Gusfield et al. 2007; Rosselló and Valiente 2009), though only a few of these incorporate the coalescent model that is central here.

By a *cycle* in either a rooted or unrooted phylogenetic network we mean a set of edges and nodes that form a cycle when all edges are treated as undirected.

#### Definition 2

Let *N* be a (rooted or unrooted) binary topological network. If no two cycles in the undirected graph of *N* share a node, then *N* is *level-1*.

In some works level-1 networks are defined as those in which no cycles share an edge; i.e., cycles are edge-disjoint rather than the stricter vertex-disjoint condition we adopt. However, in our context of binary networks these are equivalent (Rosselló and Valiente 2009).

In a level-1 network a cycle that is composed of *m* edges, (2 hybrid edges and $m-2$ tree edges) is said to be an *m*-cycle. More specifically, it is an $m_k$*-cycle* if there are exactly *k* taxa descended from its unique hybrid node (Baños 2019). This terminology can be used for semidirected networks, since ‘descended from a hybrid node’ is unambiguous, regardless of where the network is rooted.

Let *N* be an unrooted level-1 network on *X* with an *m*-cycle *C*. Then *C* induces a partition of *X* into *m* subsets according to the connected components obtained by deleting all edges in the cycle. Elements of this partition are the *blocks* of *C*. The *hybrid block of* *C* is the block of taxa descended from the hybrid node in *C*. If the blocks of *C* have $n_1,n_2,\ldots ,n_m$ taxa, then we say *C* induces a $(n_1,n_2,\ldots ,n_m)$ partition. Finally, for a cut edge $e = (v,w)$ in semidirected network, the *taxon block* below *w* is the set of taxon labels in the subgraph that contains *w* when *e* is deleted from the network.

## The Network Multispecies Coalescent Model and Quartet Concordance Factors

The Network Multispecies Coalescent (NMSC) model (Meng and Kubatko 2009) mechanistically describes the formation of gene trees within a species network, as gene lineages are traced backward in time to common ancestors in the edge populations of the network. Under it, gene trees may differ in topology from any displayed trees on the species network. Given a metric rooted phylogenetic network, the NMSC assigns positive probabilities to all resolved metric gene trees, and, through marginalization, to topological gene trees and induced gene quartet topologies.

### Definition 3

Let $$N^ + $$ be a metric rooted network on a taxon set *X*, and *A*, *B*, *C*, *D* a gene sampled from individuals in species $$a,b,c,d\in X$$ respectively. The *(scalar) quartet concordance factor* $$CF_{ab|cd} = CF_{ab|cd} (N^ + )$$ is the probability under the NMSC on $$N^ + $$ that a gene tree displays the quartet *AB*|*CD*. The *(vector) quartet concordance factor* $$\overline{CF}_{abcd} = \overline{CF}_{abcd}(N^ + )$$ is the triple

$$\begin{aligned} \overline{CF}_{abcd}=(CF_{ab|cd},CF_{ac|bd},CF_{ad|bc}) \end{aligned}$$

of concordance factors of each possible quartet on the taxa *a*, *b*, *c*, *d*.

That *CF*s for quartet networks depend only on the semidirected quartet network, was proved in Baños (2019). That result implies the following.

### Lemma 1

Under the NMSC on a level-1 network $N^+$ the values of the quartet *CF*s depend only on the induced semidirected network *N*.

Following on the first steps investigating level-1 network identifiability from quartet *CF*s taken in Solís-Lemus and Ané (2016), the next result, that most topological features of a level-1 species network are identifiable from quartet *CF*s, appeared in Baños (2019).

### Theorem 2

(Baños 2019, Theorem 4) Let *N* be a binary semidirected metric level-1 species network on taxon set *X* with $$\vert X \vert \ge 4$$. Let $$ N'$$ be the semidirected topological network obtained from *N* by contracting all 2- and 3-cycles, suppressing degree-2 nodes, and undirecting hybrid edges in 4-cycles. Under the NMSC model with generic numerical parameters, the network $$ N'$$ is identifiable from quartet *CF*s for *N*.

We take this theorem as our starting point, and in Sect. 4 focus on the remaining questions of topological identifiability: From quartet *CF*s can any aspects of 2-cycles or 3-cycles can be identified, and for 4-cycles can the hybrid node be identified? In Sect. 5 we turn to identifiability of the numerical parameters of edge lengths and hybridization probabilities. While these were not a focus in Baños (2019), partial results on local identifiability of numerical parameters were given in Solís-Lemus and Ané (2016). Note that for 4-cycle quartet networks, the map to *CF*s is overparameterized, and Gröbner basis methods easily yield the following.

### Lemma 3

Under the NMSC on a semidirected 4-taxon 4-cycle network with generic parameters, neither the hybrid node nor individual numerical parameters are identifiable from *CF*s.

### Proof

Consider the 4-taxon, 4-cycle network on taxa $Q = \{a,b,c,d\}$, with *a* the hybrid descendant, obtained from Fig. 12 (center), by setting $a=a_1$, and removing taxon $a_2$. From (Baños 2019) the hybrid node is not identifiable. But even if the hybrid node is known, the hybrid edge probabilities $h_1$, $h_2$ do not appear in the formulas for the *CFs*, so they cannot be identified. Computational algebra software (Decker et al. 2022; Grayson 2002) shows the elimination ideals retaining exactly one of the parameters $\gamma , x_1, x_2$ are generated by $CF_{ab|cd} + CF_{ac|bd} + CF_{ad|bc} - 1$. Thus no nontrivial formula relating *CF*s and a single parameter exists. $\square$

Unless explicitly stated otherwise, we assume that exactly 1 gene lineage is sampled per taxon. If 2 lineages were sampled for a taxon, say *a*, ‘pseudotaxa,’ $a_1$ and $a_2$ can be introduced by attaching a cherry leading to these at the leaf *a* of the network. Under the NMSC, *CF*s for the modified network with 1 sample from each $a_i$ are identical to those for the original network with 2 samples from *a*. Sampling more than 2 lineages per taxon only introduces new *CF*s in which 3 or 4 pseudotaxa from the same taxon appear, but due to exchangeability of lineages under the NMSC these *CF*s are always 1/3. Thus identifiability results for any multiple sampling scheme will follow from the single sample case on a modified network. No edge lengths are needed in the pseudotaxa cherries, since no coalescent event may occur on them.

Under the NMSC one can derive formulas for *CF*s for any fixed network in terms of the numerical parameters. These have the form of polynomials in the hybridization parameters $\gamma$ and the $\exp (-t)$ for all edge lengths *t*. The expression $\exp (-t)$ has a simple interpretation as the probability that two gene lineages entering an edge of length *t* coalescent units (tracing time backwards) do not coalesce within that edge. By reparameterizing using *edge probabilities* $\ell =\exp (-t)\in (0,1]$ rather than lengths $t\in [0,\infty )$, all formulas for *CF*s are given by polynomial formulas in the $\ell$s and $\gamma$s.

The $3{ n \atopwithdelims ()4}$ scalar quartet *CF*s for a fixed topological network *N* on *n* taxa then define a polynomial map from the numerical parameter space into ${\mathbb {R}}^{3{n \atopwithdelims ()4}}$. Extending the map to allow complex $\ell ,\gamma$, gives a parameterized algebraic variety. The set of multivariate polynomials in the *CF*s that vanish on the parameterization’s image is an ideal, denoted ${\mathcal {I}}(N)={\mathcal {I}}(N^+)={\mathcal {I}}(N^-)$. The zero set ${\mathcal {V}}(N)$ of the polynomials in ${\mathcal {I}}(N)$ is the Zariski closure of the parameterized variety. These notions from applied algebraic geometry provide a framework for our work. Elements of ${\mathcal {I}}(N)$ are called *invariants*, and depend only the network topology, and not its numerical parameters.

Our arguments use symbolic computations with CFs from specific networks, performed and verified by the software Singular (Decker et al. 2022) and Macaulay2 (Grayson 2002). Despite their essential role, for brevity all computational results are stated in Appendix B. That section also contains an exposition of certain linear invariants that can be derived without computation, and which simplify both computations and statements of results.

## Identifiability of Semidirected Network Topologies

### 2-Cycles

We first show 2-cycles (parallel edges) in level-1 networks are never identifiable. By *replacing* a 2-cycle with parental node *u* and child node *v* *by an edge*, or *suppressing a 2-cycle*, we mean removing its two edges, introducing a new directed edge (*u*, *v*) with a specified edge probability, and suppressing resulting nodes of degree 2.

The content of the following Lemma was essentially given in Solís-Lemus and Ané (2016), and has appeared in other works subsequently, including a generalization to 2-blobs (Ané et al. 2024, Theorem 4).

#### Lemma 4

Let $$N^ + $$ be a level-1 rooted binary metric phylogenetic network, with a 2-cycle composed of hybrid edges with edge probabilities $$ h_1,h_2$$, and corresponding hybridization parameters $$\gamma _1,\ \gamma _2=1-\gamma _1$$. Then quartet *CF*s for $$N^ + $$ under the NMSC are unchanged if the 2-cycle is replaced by an edge with edge probability $$\ell \in (0,1)$$ determined by the equation

$$\begin{aligned} 1-\ell =\gamma _1^2(1-h_1) + (1-\gamma _1)^2(1-h_2). \end{aligned}$$

Since varying the 2-cycle parameters in the above expression causes $\ell$ to range over the full interval (0, 1), we obtain the following.

#### Corollary 5

Using quartet *CF*s, under the NMSC a topological level-1 phylogenetic network *N* with a 2-cycle cannot be distinguished from the network ${\widetilde{N}}$ obtained by replacing that 2-cycle with an edge.

### 3-Cycles

**Fig. 3:**
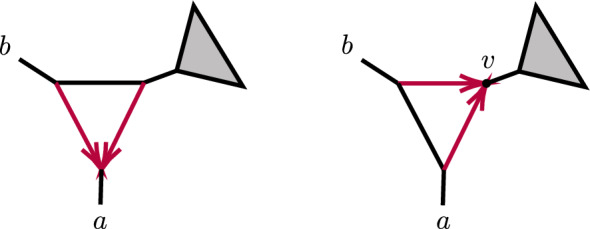
Networks with 3-cycles inducing $(1,1,n-2)$ partitions. The shaded triangle represents an arbitrary semidirected subnetwork. (left, right) correspond to cases (1, 2) of Proposition 6

The first study of whether 3-cycles on networks were detectable from *CF*s (Solís-Lemus and Ané 2016) introduced notions of “good” and “bad” triangles, corresponding to the networks in Fig. 6(right) and Figs. 3 and 5, with the terminology indicating whether the presence of a 3-cycle and partial information about its numerical parameters could be detected from *CF*s. Although we do not use these terms here, in Sect. 6 we discuss issues concerning 3-cycle inference from *CF*s relevant to that work.

Using Theorem 2, the question of identifying topological 3-cycles in a network is reduced to distinguishing between the network that theorem identifies, and networks obtained from it by replacing some set of non-cycle tree nodes with 3-cycles. We focus here on level-1 networks with 5 or more taxa, as the 4-taxon case is fully studied in Baños (2019).

#### 3-Cycles Near Leaves

We begin with a non-identifiability result, for certain 3-cycles adjacent to two pendant edges of a network, as shown in Fig. 3.

##### Proposition 6

Suppose a binary semidirected network *N* on $n\ge 4$ taxa has a 3-cycle *C* inducing a $(1,1,n-2)$ partition of the taxa. Let $N'$ be the network obtained by contracting *C* to a node. Then under the NMSC:

1. If *C* is a $3_1$-cycle, so its hybrid node has only 1 descendant taxon, the topologies of *N* and $N'$ cannot be distinguished using quartet *CF*s. That is, for any choice of parameters on one of these networks, there exist parameters on the other giving identical *CF*s. Moreover, the parameters other than those associated to *C* and internal edges adjacent to *C* may be chosen to be identical on both networks.
2. If *C* is a $3_k$-cycle with $k=n-2\ge 2$, and the parameter spaces are extended to allow all real edge lengths in the CF formulas, then for any choice of extended parameters on *N* there are extended parameters on $N'$ giving identical *CF*s, and *vice versa*. Moreover, the parameters other than those associated to *C* and internal edges adjacent to *C* may be chosen to be identical on *N* and $N'$.
  Furthermore, for strictly positive edge lengths on *N* and $N'$, there are two positive-measure subsets of parameters, $\Theta _1, \Theta _2$, for *N*, such that on $\Theta _1$ the topologies of *N* and $N'$ are not distinguishable using quartet *CF*s, and on $\Theta _2$ are distinguishable.

Note that case (1) implies that if *N* is as shown in Fig. 3(left) then *N* and $N'$ also cannot be distinguished from the network obtained from *N* by interchanging the *a* and *b* labels. In case (2), if parameters are such that *N* is not distinguishable from $N'$, then case (1) implies that they are also not distinguishable from the two networks obtained by redesignating the hybrid node in the 3-cycle to have a single descendant taxon. When *N* is distinguishable from $N'$, then by case (1) it is distinguishable from those two other networks as well.

##### Proof

Let *a*, *b* denote the taxa in the singleton blocks. For case (1), we may assume the network is rooted, with the root outside *C* and not on the pendant edges leading to *a*, *b* (Fig. 3(left)). Then under the NMSC there is a probability $p\in (0,1)$, depending on the numerical parameters of the 3-cycle, that lineages *a* and *b* fail to coalesce before leaving the 3-cycle. Replacing the 3-cycle and its adjacent edges by a 3-leaf tree where the edge leading toward the $n-2$ taxa has edge probability *p* leaves the distribution of topological gene trees, and hence quartet *CF*s, unchanged. Varying parameters over the 3-cycle or over the 3-leaf tree allows all probabilities $p\in (0,1)$ to be achieved.

**Fig. 4:**
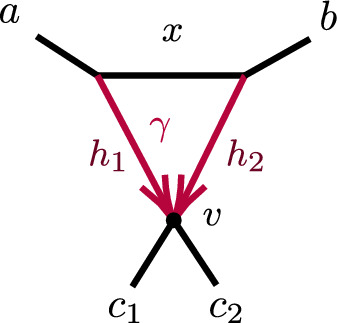
Figure for the proof of Proposition 6, case (2). A $3_2$-cycle quartet network with internal cut edge contracted to length 0, other edge probabilities $h_1,h_2,x$, and hybridization parameter $\gamma$

In case (2), let *v* denote the hybrid node in the 3-cycle, so *a*, *b* are not descendants of *v* for any rooting. (Fig. 3(right)). The value of any *CF* involving at most one of *a*, *b* is determined by the network and numerical parameters below *v*, since as a gene tree forms either a coalescent event occurs below *v*, or 3 or 4 lineages reach *v*, so that all three gene quartet topologies have probability 1/3. Thus the 3-cycle only affects values of *CF*s involving both *a* and *b*, and only through events in which no coalescence has occurred below *v*. We may thus replace the cycle and its adjacent edges to *a*, *b* with any graphical structure and parameters that produce the same probabilities of gene quartet topologies when exactly two lineages enter at *v*. These conditional probabilities are the CFs of the quartet network shown in Fig. 4:

1 $$\begin{aligned} CF_{ac|bc}= & {} \left( \gamma ^2 h_1+(1-\gamma )^2h_2 \right) /3 +\gamma (1-\gamma )\left( 1-x/3 \right) ,\nonumber \\ CF_{ab|cc}= & {} 1-2CF_{ac|bc}. \end{aligned}$$

Note that we have dropped the subscripts 1, 2 from the *c* taxa, since by exchangeability of those lineages under the NMSC, they may be assigned arbitrarily.

Now a quartet tree with topology *ab*|*cc* and internal edge probability *z* yields

$$\begin{aligned} CF_{ac|bc}=z/3,\quad CF_{ab|cc}= 1-2 CF_{ac|bc}. \end{aligned}$$

so, using (1), without changing the *CF*s the 3-cycle and edges to *a*, *b* in *N* could be replaced by a 3-leaf tree with an edge leading to an *ab* cherry having edge probability

$$\begin{aligned} z= \gamma ^2 h_1+(1-\gamma )^2h_2 +\gamma (1-\gamma )\left( 3-x \right) . \end{aligned}$$

provided $0< z < 1$. Since this inequality holds on a set of positive measure in parameter space, on that set the topologies *N* and $N'$ are not distinguishable.

However, $z>1$ also occurs on a set of positive measure. Suppose in this case that the edge $e=(v,w)$ below *v* has as its child *w* a node outside of a cycle, and let $c_1,c _2$ be taxa chosen from distinct taxon blocks below that node. Then if parameters on *N* are in the set determined by $z>1$ and the edge probability *p* for *e* satisfies $pz>1$, then for *N*

$$\begin{aligned} CF_{ac|bc}= pz/3>1/3. \end{aligned}$$

Since for a quartet tree $CF_{ac|bc}<1/3,$ *N* is distinguishable from $N'$ on this set.

If *w* is instead in a cycle, a similar argument applies. $\square$

This proof essentially follows arguments given in Baños (2019) for quartet networks with a $3_1$-cycle and $3_2$-cycle. In case (2) the parameters for which the 3-cycle is topologically identifiable are ones that make the quartet network anomalous, in the sense of Ané et al. (2024).

#### 3-Cycles on Small Networks: Algebraic Conditions

**Fig. 5:**
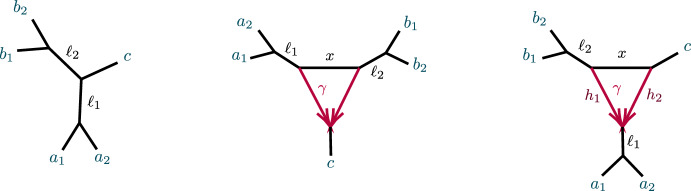
(left) The 5-taxon unrooted binary tree $$ T_5$$; (center) the 5-taxon network $$N_{5-3_1}$$ with a $$3_1$$-cycle; and (right) the 5-taxon network $$N_{5-3_2}$$ with a $$3_2$$-cycle, with numerical parameters shown. Edge probabilities of hybrid edges in $$N_{5-3_1}$$ and of pendant edges in networks are omitted, since they do not appear in formulas for the *CF*s

Figure 5 shows a 5-taxon tree, $T_5$, and two 5-taxon networks with 3-cycles, $N_{5-3_1}, N_{5-3_2}$. Propositions 32–34 of Appendix B give computational results on the ideals ${\mathcal {I}}(T_5)$, ${\mathcal {I}}(N_{5-3_1})$, and ${\mathcal {I}}(N_{5-3_2})$, showing that the polynomial

2 $$\begin{aligned} f_{abc}= 3CF_{ab|ac}CF_{ab|bc} - CF_{ab|ab} \end{aligned}$$

is in ${\mathcal {I}}(T_5)$, but not in ${\mathcal {I}}(N_{5-3_1})$ nor ${\mathcal {I}}(N_{5-3_2})$. Using expressions for CFs in terms of parameters from Proposition 32, $f_{abc}$ can be interpreted as expressing the total internal path length in the tree $T_5$ is the sum of the lengths of the two internal edges. This polynomial, and variants of it, will play an important role in identifying 3-cycles. The first result in this direction is the following.

##### Theorem 7

Under the NMSC model, the vanishing of $f_{abc}$ distinguishes a 5-taxon unrooted tree $T_5$ from the 5-taxon semidirected networks with a central 3-cycle whose contraction yields the tree $T_5$, for generic numerical parameters.

##### Proof

Consider the networks of Fig. 5 and a fourth obtained by interchanging the *a*, *b* taxa in Fig. 5 (right). Since $f_{abc}\notin {\mathcal {I}}(N)$ for the non-tree *N*, it does not vanish for all parameters on them, and is zero only for a set of measure zero in their parameter space. Thus generically the vanishing of $f_{abc}$ distinguishes $T_5$ from the others. $\square$

Propositions 32–34 also show that the two 5-taxon networks of Fig. 5 have the same associated ideals, ${\mathcal {I}}(N_{5-3_1})={\mathcal {I}}(N_{5-3_2})\subset {\mathcal {I}}(T_5)$. As a result, there is no purely algebraic means (using only polynomial equalities) of distinguishing them using *CF*s.

**Fig. 6:**
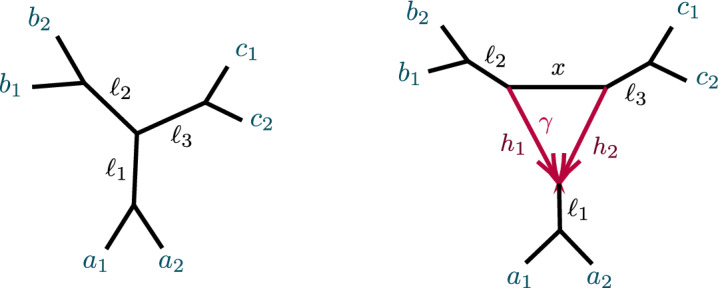
(left) The 6-taxon tree $T_6$ with three cherries. (right) The 6-taxon network $N_a$ with a central 3-cycle surrounded by 3 cherries, with $a_1,a_2$ descending from the hybrid node. The network $N_b$ is obtained by ‘rotating’ the three pairs of taxa so $b_1,b_2$ descend from the hybrid node, and similarly for $N_c$

Computational results for the 6-taxon networks $T_6$ and $N_a$ of Fig. 6 appear in Propositions 35 and 36. Note that ${\mathcal {I}}(T_6)$ contains 3 polynomials, $f_{abc}, f_{bca}, f_{cab}$, none of which are in ${\mathcal {I}}(N_a)$, expressing three different internal path length relationships in the tree. Proposition 36 implies ${\mathcal {I}}(N_a)={\mathcal {I}}(N_b)={\mathcal {I}}(N_c)$, where $N_b$ and $N_c$ differ from $N_a$ in which taxa are below the hybrid node. Thus the hybrid node of the 3-cycle in these three networks cannot be determined from purely algebraic conditions on *CF*s. While the vanishing of any of the three $f_{abc},f_{bca}, f_{cab}$ (and hence all) distinguishes the tree $T_6$ from $N_a$, $N_b,$ and $N_c$, that was already implicit in Theorem 7.

#### 3-Cycles on Small Networks: Semialgebraic Conditions

While Sect. 4.2.2 has shown the presence of a 3-cycle can be detected in some networks, that result pertains only to the undirected cycle. To obtain information on the hybrid node, we use a semialgebraic approach, focusing on polynomial inequalities.

##### Proposition 8

Let *N* be one of $T_5$, $N_{5-3_1}$, $N_{5-3_2}$ of Fig. 5, or the network $N'_{5-3_2}$ obtained from interchanging the *a*, *b* taxon labels on $N_{5-3_2}$. Let $f_{abc}$ be as in Eq. (2). Then for generic numerical parameters under the NMSC,

$$\begin{aligned} N={\left\{ \begin{array}{ll} T_5 &{} \text { if, and only if, } f_{abc}=0{,}\\ N_{5-3_2}\text { or } N'_{5-3_2} &{} \text { if } f_{abc}<0{,}\\ N_{5-3_1}, N_{5-3_2},\text { or } N'_{5-3_2} &{}\text { if } f_{abc}>0{.} \end{array}\right. } \end{aligned}$$

Moreover, $f_{abc}$ is identical on the networks $N_{5-3_2}$ and $N'_{5-3_2}$ for the same parameter values, so $f_{abc}$ gives no information to distinguish between these.

Finally, there are positive measure subsets of the numerical parameter space for $N_{5-3_2}$ on which $f_{abc}<0$ and on which $f_{abc}>0$.

##### Proof

Theorem 7 states that $f_{abc}=0$ for generic parameters if, and only if, $N=T_5$. If $N = N_{5-3_1}$ then using the formulas for *CF*s in Proposition 33 gives, for $\gamma ,x, \ell _1, \ell _2 \in (0,1)$,

$$\begin{aligned} f_{abc}&= [\ell _1\ell _2 (\gamma + x-\gamma x)(1-\gamma + \gamma x) -\ell _1\ell _2x]/3 \\&=\ell _1\ell _2\gamma (1-\gamma )(x-1)^2/3>0. \end{aligned}$$

Since $f_{abc}$ is invariant under interchanging the *a*s and *b*s, its values for $N_{5-3_2}$ and $N'_{5-3_2}$ are the same.

Specific examples of parameters on $N_{5-3_2}$ show both $f_{abc}<0$ and $f_{abc}>0$ can occur, and by continuity there are positive measure subsets of parameter space on which these occur. $\square$

If a 5-taxon network does have a 3-cycle *C*, then this proposition may provide some information on the hybrid node’s location. For instance, $f_{abc}<0$ implies the taxon *c* which is not in a cherry on the tree obtained by contracting *C* to a vertex is also not a hybrid descendant of the 3-cycle. However, for other numerical parameters $f_{abc} > 0$, in which case there is no information on the hybrid location.

To further develop semialgebraic tests for 3-cycle hybrid nodes, we again consider the 6-taxon networks $N_a,N_b,N_c$ described in Fig. 6. Define the following functions of the *CF*s, building on the $f_{xyz}$:

3 $$\begin{aligned} G_{abc}&=-f_{abc}CF_{ac|bc}+2 f_{bca}C_{ab|ac} -f_{cab}C_{ab|bc}\nonumber \\&=CF_{ac|ac}CF_{ab|bc}-2CF_{bc|bc}CF_{ab|ac} + CF_{ab|ab}CF_{ac|bc},\nonumber \\ G_{cab}&=CF_{bc|bc}CF_{ab|ac}-2CF_{ab|ab}CF_{ac|bc} + CF_{ac|ac}CF_{ab|bc}\nonumber ,\\ G_{bca}&=CF_{ab|ab}CF_{ac|bc}-2CF_{ac|ac}CF_{ab|bc} + CF_{bc|bc}CF_{ab|ac}. \end{aligned}$$

Note that $G_{xyz}\in {\mathcal {I}}(T_6)$, $G_{xyz}=G_{xzy}$ and $G_{abc} + G_{cab} + G_{bca}=0$.

##### Proposition 9

Under the NMSC, for *CF*s arising from the tree $T_6$, $G_{xyz}=0$ for all *x*, *y*, *z*, while $G_{xyz}>0$ for *CF*s arising from the network $N_x$.

If a network is known to have one of the topologies $N_a,N_b,N_c$, then at least one of these topologies can be ruled out by the signs of $G_{abc},G_{cab},G_{bca}$: If $G_{xyz}<0$ then the network is not $N_x$.

Finally, there are positive measure subsets of the numerical parameter space for $N_y$ and $N_z$ on which $G_{xyz}<0$ and on which $G_{xyz}>0$.

##### Proof

That $G_{xyz}=0$ for $T_6$ restates that $G_{xyz}\in {\mathcal {I}}(T_6)$. Using formulas from Proposition 36, for *CF*s from $N_a$,

$$\begin{aligned} 9\, G_{abc}&={9(CF_{ac|ac}CF_{ab|bc}-2CF_{bc|bc}CF_{ab|ac} + CF_{ab|ab}CF_{ac|bc})}\\&=\ell _1\ell _3(\gamma ^2h_1x + \gamma ^2h_2-2\gamma ^2-2\gamma h_2 + 2\gamma + h_2) \ell _2(x + \gamma -\gamma x) \\&\quad -2x\ell _2\ell _3 \ell _1(\gamma ^2h_1 + \gamma ^2h_2 + \gamma ^2x-3\gamma ^2-2\gamma h_2-\gamma x + 3\gamma + h_2)\\&\quad + \ell _1\ell _2(\gamma ^2h_1 + \gamma ^2h_2x-2\gamma ^2-2\gamma h_2x + 2\gamma + h_2x) \ell _3(\gamma x-\gamma + 1)\\&=\gamma (1-\gamma )(1-x)^2\ell _1\ell _2\ell _3 [h_1\gamma + h_2(1-\gamma ) + 2]>0. \end{aligned}$$

Since $G_{abc}+G_{cab}+G_{bca}=0$ and one of these terms is positive for each of $N_a,N_b,N_c$, at least one is negative.

One can find specific parameters on $N_y$ for which $G_{xyz}<0$ and $G_{xyz}>0$, and by continuity these conditions hold on sets of positive measure. $\square$

**Fig. 7:**
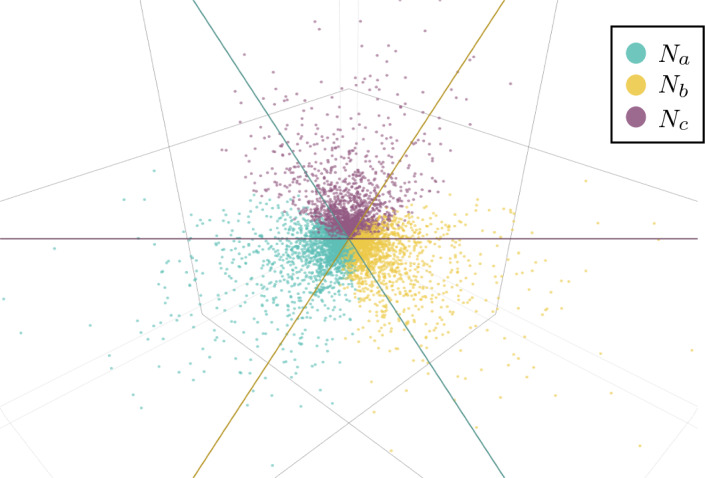
Values of $(G_{abc}, G_{bca},G_{cab})$ plotted in three dimensions, for random numerical parameter values on each of the three networks $N_a,N_b,N_c$. Color indicates network topology. Plotted points lie in the plane $x+y+z=0$, which is viewed orthogonally. The three coordinate planes $x=0,y=0,z=0$ intersect this plane in the colored lines, separating the points by color into overlapping half-planes. Numerical parameters for networks were chosen uniformly from the interval [0, 1] (Color figure online)

Figure 7 illustrates the proposition, showing $(G_{abc}, G_{bca},G_{cab})$ for randomly chosen numerical parameters on each of the networks $N_a,N_b,N_c$, with color indicating the network topology. Since the points lie in a plane *P* through the origin, the axes have been rotated to view the plane orthogonally. The three planes $G_{xyz}=0$ intersect *P* in lines which divide the plot into six sectors. On three of these sectors exactly one color appears, indicating that the network topology is determined by the positivity of exactly one $G_{xyz}$. On the 3 sectors where two colors appear, two of the $G_{xyz}$ are positive, so only one of the network topologies is ruled out.

##### Remark 1

It is natural to ask if $f_{xyz}$ or $G_{xyz}$ could be used to detect 3-cycles in situations where incomplete lineage sorting is negligible, so that all gene trees are displayed on the species network. This scenario is modeled by immediate coalescence of gene lineages on entering a common network edge or, equivalently, by a limiting model of the NMSC, in which all edge probabilities go to 0. (See Allman et al. 2022, Sect. 6.2 for more details.) The formulae for *CF*s given in this work still apply with all edge probabilities set to 0, and one finds that for all the 5-taxon networks of Proposition 8$f_{abc}=0$, while for all the 6-taxon networks of Proposition 9$G_{xyz}=0$. Indeed, these functions depend only on *CF*s for quartet trees *not* displayed on the networks, which are therefore all zero.

Another model of interest, the common inheritance coalescent model (Gerard et al. 2011), gives only gene trees arising from the coalescent process on the displayed trees of the species network, with the probability of each displayed tree the product of its edges’ hybridization parameters. For this model, the functions $f_{abc},G_{xyz}$ are generically non-zero, and produce a figure similar to Fig. 7 (calculations and figure not shown). Although our investigation of identifiability of that model and its generalization to the correlated inheritance coalescent model (Fogg et al. 2023) are not complete, this illustrates how a coalescent model of some sort allows for greater identifiability of network structure.

Proposition 9 and Fig. 7 suggest determining which of $N_a,N_b,$ or $N_c$ produced certain numerical *CF*s may be impossible, which we rigorously show by the following example.

##### Example 1

(Non-identifiability of the hybrid node in a 3-cycle) Consider the network $N_a$ with parameters

$$\begin{aligned} \gamma ^{(a)}= & {} \frac{28}{100},\quad h_1^{(a)}=\frac{83}{100}, \quad h_2^{(a)}=\frac{78}{100}, \quad x^{(a)}=\frac{98}{100},\\ \ell _1^{(a)}= & {} \frac{88}{100}, \quad \ell _2^{(a)}=\frac{61}{100}, \quad \ell _3^{(a)}=\frac{50}{100}, \end{aligned}$$

and the network $N_b$ with parameters

$$\begin{aligned} \gamma ^{(b)}&=\frac{236700}{253367}, \quad h_1^{(b)}=\frac{84456638}{87243675}, \quad h_2^{(b)}=\frac{27286250}{31593489}, \\ x^{(b)}&=\frac{2722883}{2976250}, \quad \ell _1^{(b)}=\frac{809409}{1315000}, \quad \ell _2^{(b)}=\frac{1}{2}, \quad \ell _3^{(b)}=\frac{26191}{31250}, \end{aligned}$$

where the parameters for $N_b$ are as shown for $N_a$ in Fig. 6 but with taxon labels $(a_1,a_2)$, $(b_1,b_2)$ and $(c_1,c_2)$ replaced by $(b_1,b_2)$, $(c_1,c_2)$ and $(a_1,a_2)$ respectively. Then the *CF*s of $N_a$ and $N_b$ are equal. Specifically, for both $N_a$ and $N_b$,

$$\begin{aligned} CF_{bc|bc}&= \frac{2989}{30000}, \quad CF_{ab|ab}=\frac{906412969}{5859375000} ,\quad CF_{ac|ac}=\frac{29951713}{234375000}, \\ CF_{ab|ac}&=\frac{602701}{2343750} \quad CF_{ab|bc}=\frac{9394}{46875}, \quad CF_{ac|bc}=\frac{1243}{7500}. \end{aligned}$$

In fact, there is a neighborhood in ${\mathcal {V}}(N_a)={\mathcal {V}}(N_b)$ of the *CF* point of this example contained in the image of the parameterizations of both $N_a$ and $N_b$. Indeed, a computation of the Jacobians for the two parameterization maps at the example parameters shows that locally the images are of dimension 6, which matches the dimension of the variety. A sufficiently small neighborhood of the *CF* point is thus in the image of the parameterizations for both $N_a$ and $N_b$, with inverse images of positive measure. One may similarly show, using a *CF* point that arises only from $N_a$ (lying in a uniformly colored sector in Fig. 7), that there is a set of positive measure in the $N_a$ parameter space which gives *CF*s in the image of the parametrization of $N_a$ only. We combine these results formally in the following theorem.

##### Theorem 10

There exists a positive measure subset of the numerical parameter space of $N_a$ for which it is distinguishable from $T_6$, $N_b$, and $N_c$, and a positive measure subset of the parameter space for which only the undirected network can be distinguished from $T_6$, with 1 node in the 3-cycle determined to be non-hybrid.

Again using the parameter values in Example 1, an analog of this result for 5-taxon networks with a single 3-cycle can be established.

##### Theorem 11

There exist positive measure subsets of the numerical parameter spaces of $N_{5-3_1}$ and $N_{5 -3_2}$ for which the semidirected network topologies are distinguishable from the other networks among $T_5,N_{5-3_1}, N_{5-3_2}, N'_{5-3_2},$ and positive measure subsets of the parameter spaces for which they are not distinguishable from at least one other of $N_{5-3_1}, N_{5-3_2}, N'_{5-3_2}$.

##### Proof

First, suppose the network is $N_{5-3_2}$. Dropping a taxon to pass to a quartet network with a $3_2$-cycle, Proposition 6 implies that the semidirected topology is identifiable on some positive measure subset of parameters. That there is such a set on which the semidirected topology is not identifiable follows from using the parameter values of Example 1 (after dropping an appropriately chosen taxon) on such networks with different hybrid cherries, and computing Jacobians to verify that an open set of such examples exists.

To investigate identifiability for the network $N_{5-3_1}$, consider the function

4 $$\begin{aligned} {\tilde{f}}=f_{abc}-(1/2)CF_{ab|ab}. \end{aligned}$$

We first show that ${\tilde{f}} <0$ for all parameters on $N_{5-3_2}$. Using Proposition 34 to expand in terms of parameters,

$$\begin{aligned} {\tilde{f}}= & {} \ell _1\ell _2[ (\gamma ^2h_1 + \gamma (1-\gamma )(3-x)+ (1-\gamma )^2h_2) (\gamma + (1-\gamma ) x)/3\\{} & {} {} -(\gamma ^2h_1 + 2\gamma (1-\gamma )+ (1-\gamma )^2 h_2x)/2]. \end{aligned}$$

Since $h_1$ appears linearly in this expression with a negative coefficient, we set $h_1=0$ to bound ${\tilde{f}}$ above. The coefficient of $h_2$, which also appears linearly, may be positive or negative, so we consider $h_2=0$ and 1. If $h_2=0$,

$$\begin{aligned} {\tilde{f}}&=\ell _1\ell _2\left[ ( \gamma (1-\gamma )(3-x)) (\gamma + (1-\gamma ) x)/3 -\gamma (1-\gamma )\right] \\&=-\ell _1\ell _2\gamma (1-\gamma )\left[ 3(1-x)(1-\gamma )+x(\gamma + (1-\gamma ) x)\right] /3<0, \end{aligned}$$

while if $$h_2=1$$,

$$\begin{aligned} {\tilde{f}}&=\ell _1\ell _2 \left[ ( \gamma (1-\gamma )(3-x)+ (1-\gamma )^2) (\gamma + (1-\gamma ) x)/3-( 2\gamma (1-\gamma )\right. \\&\quad \left. +(1-\gamma )^2 x)/2\right] \\&=-\ell _1\ell _2(1-\gamma ) \left[ \gamma x(\gamma + (1-\gamma ) x)+ (1-\gamma )\left( 2 \gamma (1-x) + x/2\right) \right] /3<0. \end{aligned}$$

It is easy, however, to find an open set of parameters for $N_{5-3_1}$ for which ${\tilde{f}}>0$, and on that set *c* is identifiable as the hybrid block.

We obtain a set on which the semidirected topology of $N_{5-3_1}$ is not identifiable by again using the parameter values in Example 1. $\square$

#### Large Networks with 3-Cycles

**Fig. 8:**
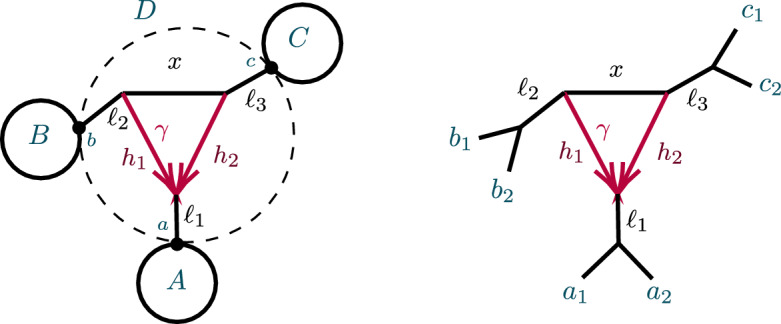
(left) A decomposition of a level-1 network $N^ +$ with a 3-cycle into 4 subnetworks, denoted *A*, *B*, *C*, *D*, with root in *C*. (right) The semidirected 3-cycle network $N_{6-3_2}$ with 3 cherries, which is a simple instance of the network on the left

After considering specific 5- and 6-taxon networks with a single 3-cycle, we shift focus to 3-cycles in general networks $$N^ + $$. We extend the previous results on semialgebraic identifiability of both cycles and hybrid nodes, using a decomposition of $$N^ + $$ into 4 subnetworks, as in Fig. 8. A similar decomposition is used in Gross et al. (2023), of a level-1 network into trees and ‘sunlets,’ but that work does not model coalescence, so the details are quite different. Our decomposition extends to larger cycles but we present only the 3-cycle case needed here.

The subnetworks in Fig. 8 are:

*D*: The 3-cycle and its three adjacent cut edges, with pendant vertices *a*, *b*, *c*, where *a* is the child of the hybrid node of the cycle;
*A*, *B*, *C*: The connected components containing *a*, *b*, *c*, respectively, when the edges and internal nodes of *D* are deleted from $$N^ + $$.

Note that *a*, *b*, *c* are each in two of these subnetworks. Since the root must be above *D*’s hybrid node, and the semidirected network is unchanged by moving the root along tree edges, we may assume the root lies in *B* or *C*, and, after renaming, in *C*.

The *CF* of any quartet under the NMSC on $$N^ + $$ has an algebraic decomposition into terms associated to the subnetworks *A*, *B*, *C*, *D*, which we next develop. We use two facts about coalescent events between 4 lineages leading to gene quartets:

1. The first coalescent event between 2 of the lineages determines the gene quartet tree that forms, and
2. Conditioned on 3 or 4 lineages reaching a common node with no previous coalescence, by exchangeability of lineages each quartet has probability 1/3.

For $S\in \{A,B,C,D\}$ and a gene quartet *xy*|*zw* where $x,y,z,w\in X$ are taxa on $N^+$, we define an event, denoted ${\mathcal {C}}_S \rightarrow xy|zw,$ that captures whether the behavior of gene lineages in *S* ensures that under the coalescent model the gene tree *xy*|*zw* is formed, or will be, with a determined probability. This may be due to a coalescent event occurring in *S*, or 3 or 4 lineages reaching a common node in *S* without having yet coalesced. Since any coalescent event between any of the four lineages that occurs below *S* would already determine the quartet tree, we define events conditional on the lineages from those of $\{x,y,z,w\}$ that are below *S* not having coalesced below *S*. For instance, in Fig. 8(left) if *S* is *D* and the quartet of interest is $\{a_1, b_1, b_2, c_1\}$, with $a_1$ on *A*, $b_1,b_2$ on *B*, and $c_1$ on *C* then we condition on $b_1,b_2$ not having coalesced in *B*. More formally, consider the following events, described backwards in time:

$$\begin{aligned} E={E(S,\{x,y,z,w\} })&= \text {No coalescence between any of the lineages}\, x,y,z,w\\&\qquad \text {that may enter}\, S\, \text {occurs below their entry to}\,S.\\ F=F(S,xy|zw)&=\text {There is a node}\, v\,\text { in}\, S\, \text {that 3 or 4 of the}\, x,y,z,w\, \text {lineages reach }\\&\qquad \text {with no coalescence occurring below}\, v,\, \text {and then}\, xy|zw\,\text { forms.}\\ G=G(S,xy|zw)&= \text {A first coalescence occurs at some node}\, v\, \text {in}\, S\,\text { so that }\\&\qquad xy|zw\, \text {forms, without 3 or 4 lineages having reached a }\\&\qquad \text {common node below}\, v. \end{aligned}$$

Then $${\mathcal {C}}_S \rightarrow xy|zw$$ denotes $$(F\cup G)|E$$.

Let $P({\mathcal {C}}_S \rightarrow xy|zw)$ denote the conditional probability of the event ${\mathcal {C}}_S \rightarrow xy|zw$. Then with $a_i,b_i,c_i$ distinct taxa from *A*, *B*, *C*, respectively, a few example decompositions of *CF*s are:

$$\begin{aligned} CF_{a_1a_2|a_3 b_1}&=P({\mathcal {C}}_{A}\rightarrow a_1a_2|a_3b_1),\\ CF_{a_1a_2|b_1 c_1}&=P({\mathcal {C}}_A\rightarrow a_1a_2|b_1 c_1) + (1- P({\mathcal {C}}_A\rightarrow a_1a_2|b_1 c_1) )P({\mathcal {C}}_{D}\rightarrow a_1a_2|b_1 c_1),\\ CF_{a_1a_2|c_1 c_2}&=P({\mathcal {C}}_A\rightarrow a_1a_2|c_1 c_2) + (1-P({\mathcal {C}}_A\rightarrow a_1a_2|c_1 c_2) )P({\mathcal {C}}_{D}\rightarrow a_1a_2|c_1 c_2) \\&\quad + (1-P({\mathcal {C}}_A\rightarrow a_1a_2|c_1 c_2) )(1-P({\mathcal {C}}_D\rightarrow a_1a_2|c_1 c_2) )P({\mathcal {C}}_C\rightarrow a_1a_2|c_1 c_2). \end{aligned}$$

For calculating probabilities associated to *D*, we suppress indices on taxa. This is allowable since, conditioned on distinct lineages entering *D*, $a_1,a_2$ are exchangeable, as are $b_1,b_2$. Thus, for instance,

$$\begin{aligned} P({\mathcal {C}}_D\rightarrow {ab|bc})=P({\mathcal {C}}_D\rightarrow {a_1b_1|b_2c_1})=P({\mathcal {C}}_D\rightarrow {a_2b_2|b_1c_2}). \end{aligned}$$

Significantly, all *CF*s for $N^+$ can be computed using only the following probabilities associated to *D* together with expressions dependent only on *A*, *B*, *C*:

$$\begin{aligned} p_1=P({\mathcal {C}}_D\rightarrow ab|cc)&=1-\ell _3( 1-\gamma +\gamma x), \\ p_2=P({\mathcal {C}}_D\rightarrow aa|cc)&= 1-\ell _1\ell _3(\gamma ^2h_1x+2\gamma (1-\gamma ) +(1-\gamma )^2h_2),\\ p_3=P({\mathcal {C}}_D\rightarrow bb|cc)&=1-x\ell _2\ell _3,\\ p_4=P({\mathcal {C}}_{D}\rightarrow ab|bc)&=\ell _2\left( \gamma + (1 - \gamma ) x \right) /3 {,}\\ P({\mathcal {C}}_{D}\rightarrow bb|ac)&=1-2p_4,\\ p_5=P({\mathcal {C}}_{D}\rightarrow ab|ac)&= \ell _1(\gamma ^2h_1 + \gamma (1-\gamma )(3-x) + (1-\gamma )^2 h_2 )/3,\\ P({\mathcal {C}}_{D}\rightarrow aa|bc)&=1-2 p_5,\\ p_6=P({\mathcal {C}}_{D}\rightarrow ab|ab)&= \ell _1\ell _2(\gamma ^2h_1 + 2\gamma (1-\gamma ) + (1-\gamma )^2 h_2x)/3,\\ P({\mathcal {C}}_{D}\rightarrow aa|bb)&=1-2 p_6. \end{aligned}$$

The 6 linearly independent polynomials, $p_1,p_2,\ldots , p_6$ parameterize a variety, ${\mathcal {V}}(D)$. Combined with the previous discussion of decomposing *CF* formulas, this yields the following.

##### Proposition 12

Let ${\mathcal {V}}_{ N}$ be the *CF* variety for a semidirected network (not necessarily level-1) *N* with the form shown in Fig. 8, and numerical parameter space $\Theta (N)=\Theta _{A,B,C}\times \Theta _D$. Let ${\mathcal {V}}_D$ denote the Zariski closure of the image of the parameterization $\phi :{\mathbb {C}}^7\rightarrow {\mathbb {C}}^6$, defined by

$$\begin{aligned} \phi (\gamma ,\ell _1,\ell _2,\ell _3,h_1,h_2,x)=(p_1,p_2,p_3,p_4,p_5,p_6), \end{aligned}$$

with the $p_i$ given above. Then the map $CF: \Theta (N)\rightarrow {\mathbb {C}}^{3{n\atopwithdelims ()4}}$ factors as

5 $$\begin{aligned} CF: \Theta ( N) =\Theta _{A,B,C}\times \Theta _D \xrightarrow {\pi \times \phi }\Theta _{A,B,C}\times {\mathcal {V}}_D\rightarrow {\mathcal {V}}_N\subset {\mathbb {C}}^{3{n\atopwithdelims ()4}}. \end{aligned}$$

where $\pi$ is the map projecting $\Theta (N)$ onto the numerical parameters on *A*, *B*, *C* only.

Proposition 37 shows that ${\mathcal {V}}_D={\mathbb {C}}^6$, and thus $\phi$ is an infinite-to-1 map, establishing the following.

##### Corollary 13

Consider a semidirected topological network *N* with a 3-cycle, with decomposition as in Fig. 8(left). Then no test using polynomial equalities in quartet *CF*s can identify the hybrid node in the 3-cycle.

Specifically, if *N*’s root must be in the subnetwork *C* because of the semidirected topology of *C*, then the network $N_B$ which has *A*, *B* interchanged from $N=N_A$, so that *B* is below the 3-cycle’s hybrid node, leads to the same ideal of invariants, that is, ${\mathcal {I}}(N_A)={\mathcal {I}}(N_B)$. If the semidirected topology of *N* allows for rooting in either subnetwork *B* or *C*, then ${\mathcal {I}}(N_A)={\mathcal {I}}(N_B)= {\mathcal {I}}(N_C)$.

##### Proof

If deleting the 3-cycle from the network induces a $(n_1,n_2,n_3)$ partition of the taxa with all $n_i\ge 2$, then the corollary follows directly from Proposition 12 and Proposition 37. Cases with $n_i=1$ then follow by deleting taxa from an appropriate network with all $n_i\ge 2$, intersecting the ideals with a ring generated by fewer CFs. $\square$

Note that this corollary applies to networks with more than one 3-cycle. However, when multiple cycles are present, the location of one cycle’s hybrid node indicates that one of the nodes in a descendant cycle cannot be hybrid. Thus for a network with *k* 3-cycles, there are between $2^k$ and $3^k$ networks differing only in the choice of hybrid nodes in the 3-cycles, all of which are algebraically indistinguishable using *CF*s.

Nonetheless, using semialgebraic tests, we can obtain additional information on hybrid node location, as the following generalization of Proposition 9 shows.

##### Proposition 14

Consider a partition of a taxon set *X* into three blocks of size at least 2. For any network *N* (not necessarily level-1) with a node or 3-cycle inducing these blocks, denote the node or 3-cycle and its adjacent edges by *D*, and the subgraphs attached to *D* as *A*, *B*, *C* (as in Sect. 4.2.4 for a cycle).

Let $G_{abc},\ G_{cab},\ G_{bca}$ be as defined by Eq. (3), for any distinct taxa $a_i$ on *A*, $b_i$ on *B*, and $c_i$ on *C*. Then *D* is:

$$\begin{aligned} {\left\{ \begin{array}{ll} \text{ a } \text{3-leaf } \text{ tree } &{}{} \text{ if }\quad G_{abc}=G_{bca}=G_{cab}=0,\\ \text{ a } \text{3-cycle } \text{ and } \text{ adjacent } \text{ edges } &{}{} \text{ if }\quad G_{xyz}>0,\ G_{yzx}\le 0,\ G_{zxy}\le 0 \\ ~ \text{ with }\, x\, \text{ below } \text{ the } \text{ hybrid } \text{ node } &{}{\quad } \text{ for }~ \{x,y,z\}=\{a,b,c \}{,}\\ \text{ a } \text{3-cycle } \text{ and } \text{ adjacent } \text{ edges } \text{ with } &{}{} \text{ if }\quad G_{xyz}>0,\ G_{yzx}> 0,\ G_{zxy}< 0 \\ ~ \ \ x\,\text{ or }\, y\, \text{ below } \text{ the } \text{ hybrid } \text{ node } &{}{\quad }\text{ for }~ \{x,y,z\}=\{a,b,c \}. \end{array}\right. } \end{aligned}$$

Moreover, for a network *N* with a 3-cycle *D* and descendants of *x* forming its hybrid block, there exist positive measure subsets of parameters on *D* on which $G_{yzx}$ and $G_{zxy}$ satisfy both of the above sign conditions.

##### Proof

First suppose *D* is a 3-cycle and, without loss of generality, *A* is below the hybrid node. Then we decompose formulas for *CF*s for *N* as

$$\begin{aligned} CF_{ab|ab}&=(1-P({\mathcal {C}}_A\rightarrow aa|bb)) (1-P({\mathcal {C}}_B\rightarrow aa|bb)) P({\mathcal {C}}_D\rightarrow ab|ab), \\ CF_{ac|bc}&= (1-P({\mathcal {C}}_D\rightarrow ab|cc))P({\mathcal {C}}_C\rightarrow ac|bc),\\ CF_{ac|ac}&=(1-P({\mathcal {C}}_A \rightarrow aa|cc)) (1-P({\mathcal {C}}_D\rightarrow aa|cc))P({\mathcal {C}}_C\rightarrow ac|ac),\\ CF_{ab|bc}&=(1-P({\mathcal {C}}_B \rightarrow ac|bb)) P({\mathcal {C}}_D \rightarrow ab|bc),\\ CF_{bc|bc}&=(1-P({\mathcal {C}}_B \rightarrow bb|cc)) (1-P({\mathcal {C}}_D\rightarrow bb|cc))P({\mathcal {C}}_C\rightarrow bc|bc),\\ CF_{ab|ac}&= (1-P({\mathcal {C}}_A \rightarrow aa|bc)) P({\mathcal {C}}_D \rightarrow ab|ac). \end{aligned}$$

Since

$$\begin{aligned} P({\mathcal {C}}_A\rightarrow aa|bb)&=P({\mathcal {C}}_A \rightarrow aa|cc)=P({\mathcal {C}}_A \rightarrow aa|bc),\\ P({\mathcal {C}}_B\rightarrow aa|bb)&=P({\mathcal {C}}_B \rightarrow bb|cc)=P({\mathcal {C}}_B \rightarrow ac|bb),\\ P({\mathcal {C}}_C\rightarrow ac|bc)&=P({\mathcal {C}}_C\rightarrow ac|ac)=P({\mathcal {C}}_C\rightarrow bc|bc), \end{aligned}$$

it follows that

$$\begin{aligned} G_{abc}(N)= & {} (1-P({\mathcal {C}}_A\rightarrow aa|bb) )(1-P({\mathcal {C}}_B\rightarrow aa|bb)) P({\mathcal {C}}_C\rightarrow ac|ac)\, \\{} & {} {} \times \bigg [(1-P({\mathcal {C}}_D\rightarrow aa|cc) ) P({\mathcal {C}}_D\rightarrow ab|bc)\\{} & {} {\quad } -2 (1-P({\mathcal {C}}_D\rightarrow bb|cc)) P({\mathcal {C}}_D\rightarrow ab|ac) \\{} & {} {\quad } + P({\mathcal {C}}_D\rightarrow ab|ab)(1-P({\mathcal {C}}_D\rightarrow ab|cc))\bigg ]. \end{aligned}$$

But the terms in the last factor, all of which depend only on *D*, arise as multiples of *CF*s on the network $N_{6-3_2}$ of Fig. 8(right),

$$\begin{aligned} 1- P({\mathcal {C}}_D\rightarrow aa|cc)&= 3CF_{ac|ac}(N_{6-3_2}),\qquad&P({\mathcal {C}}_D\rightarrow ab|bc)&=CF_{ab|bc}(N_{6-3_2}),\\ 1- P({\mathcal {C}}_D\rightarrow bb|cc)&=3CF_{bc|bc}(N_{6-3_2}), \qquad&P({\mathcal {C}}_D\rightarrow ab|ac)&=CF_{ab|ac}(N_{6-3_2}),\\ P({\mathcal {C}}_D\rightarrow ab|ab)&=CF_{ab|ab}(N_{6-3_2}), \qquad&1-P({\mathcal {C}}_D\rightarrow ab|cc)&=3CF_{ac|bc}(N_{6-3_2}). \end{aligned}$$

Thus, by Proposition 9, $G_{abc}(N)=$

$$\begin{aligned} 3(1-P({\mathcal {C}}_A\rightarrow aa|bb) )(1-P({\mathcal {C}}_B\rightarrow aa|bb)) P({\mathcal {C}}_C\rightarrow ac|ac)G_{abc}(N_{6-3_2})>0. \end{aligned}$$

Since $G_{abc}+G_{cab} +G_{bca}=0$, either 1 or 2 of these terms are positive, and the two cases for 3-cycle *D*s follow. The case of the network *N* with *D* a node is obtained by setting $x=h_1=h_2=0$ in the formulas for any of the 3-cycle networks, showing, for instance, that $G_{abc}(N)$ is a multiple of $G_{abc}(T_6)=0.$

The final statement on positive measure subsets of parameter space follows from Proposition 9. $\square$

Proposition 14 yields the following generalization of Theorem 10.

##### Theorem 15

Consider a partition of a taxon set *X* into three blocks of size at least 2. Then for all networks (not necessarily level-1) with a node or 3-cycle inducing these blocks, the presence of the node or the (undirected) 3-cycle along with one non-hybrid block is identifiable. If the network has a 3-cycle then there are positive measure subsets of its parameter space on which the hybrid node can be determined, and on which it cannot.

##### Proof

By Proposition 14, an undirected 3-cycle is signaled by the non-vanishing of at least one of $G_{abc}$, $G_{bca}$ or $G_{cab}$, and for a 3-cycle, a non-hybrid block is identifiable since one of the *G*s must be negative. That the hybrid node can be identified on a positive measure set follows from the existence of such a set for which only one *G* is positive. That the hybrid node cannot be identified on another set is seen by choosing specific parameters on 3-cycles with different hybrid nodes (e.g., using parameters given in Example 1 for the 3-cycle and adjacent edge parameters) which produce the same values for the $p_i$. $\square$

If $n_i=1$ for some *i*, then similar arguments as given for Proposition 14 and Theorem 15 shows the function $f_{xyz}$ can identify the presence of a 3-cycle, but possibly not its hybrid node. While we omit the proof, we state the result.

##### Proposition 16

Consider a partition of a taxon set *X* into three blocks of size $1, n_1,n_2$ with $n_i\ge 2$. For any network *N* (not necessarily level-1) with a node or 3-cycle inducing these blocks, let *D* denote the node or 3-cycle and adjacent edges, and *A*, *B*, *C* the subgraphs attached to *D* by the adjacent edges, with *C* being a single node. Let $f_{abc}$ be as in Proposition 8, for any distinct $a_i$ on *A*, $b_i$ on *B*, and *c* on *C*. Then for generic numerical parameters, *D* is:

$$\begin{aligned} {\left\{ \begin{array}{ll} \text {a 3-leaf tree} &{} \text { if, and only if, }f_{abc}=0,\\ \text {a 3-cycle and adjacent edges with}\\ \ \ A\,\text { or}\, B\,\text { below the hybrid node}&{}\text { if }f_{abc}<0,\\ \text {a 3-cycle and adjacent edges with} \\ \ \ A, B,\, \text {or}\, C\,\text { below the hybrid node}&{}\text { if }f_{abc}>0. \end{array}\right. } \end{aligned}$$

Finally, there are positive measure subsets of the numerical parameter space for the networks with a 3-cycle *D* and either *A* or *B* below its hybrid node on which $f_{abc}<0$ and on which $f_{abc}>0$.

For identifying the hybrid node in a 3-cycle inducing a $(1,n_1,n_2)$ partition when there is a single descendant of the hybrid node, we generalize Theorem 11.

##### Theorem 17

Consider a partition of a taxon set *X* into three blocks of sizes $1,n_1,n_2$ with $n_i\ge 2$. Then for all networks (not necessarily level-1) with a 3-cycle inducing these blocks, there are positive measure subsets of the parameter space on which the hybrid node of the 3-cycle is identifiable, and on which it is not.

##### Proof

Let ${\tilde{f}}$ be as defined in Eq. (4). We first show the result for a general network *N* with a 3-cycle with a single hybrid descendant. For such a network, using decompositions as in Sect. 4.2.4 but with *C* a hybrid singleton taxon and the root in *B*, we find that for any choices of two taxa in the *A* and *B* blocks

$$\begin{aligned} {\tilde{f}}(N)=3(1-P(C_A\rightarrow aa|bc))P(C_B\rightarrow ab|bc){\tilde{f}}(N_{5-3_1}), \end{aligned}$$

where $N_{5-3_1}$ is given parameters from the 3-cycle and adjacent edges of *N*. Similarly, if a non-hybrid block *C* is the singleton

$$\begin{aligned} {\tilde{f}}(N)=3(1-P(C_A\rightarrow aa|bc))P(C_B\rightarrow ab|bc){\tilde{f}}(N_{5-3_2}), \end{aligned}$$

where $N_{5-3_2}$ is given parameters from the 3-cycle and adjacent edges of *N*. Thus the signs of ${\tilde{f}}$ on *N* can be used as in the proof of Theorem 11 to obtain the claim when the hybrid block is a singleton.

If the singleton block is not hybrid on *N* the claim is established as for Theorem 11, by passing to a subnetwork with a $3_2$-cycle and using the parameters of Example 1. $\square$

Finally, if a 3-cycle induces a $(1,1,n-2)$ partition then Proposition 6 applies directly to analyze identifiability.

### 4-Cycles

**Fig. 9:**
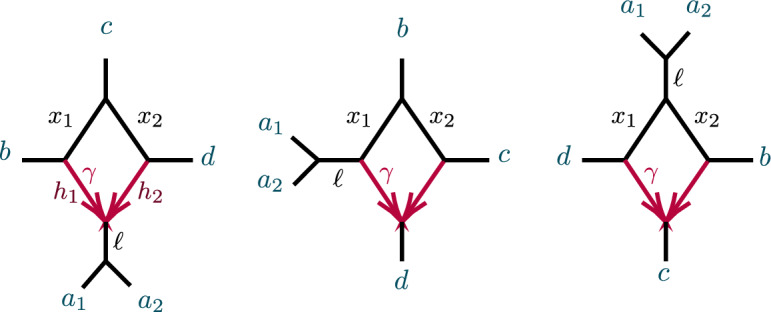
The semidirected 5-taxon binary networks with a single 4-cycle, up to taxon labelling. We denote these by $N_s$, $N_w$, $N_n$ from left to right, according to compass directions for the $a_1,a_2$ cherry when the hybrid node is located at south. Note that $N_e$ is omitted since, up to taxon labelling, it is the same as $N_w$. Edge probabilities and the hybridization parameter $\gamma$ are shown next to edges

To study topological 4-cycle identifiability beyond the results of Solís-Lemus and Ané (2016) and Baños (2019), we consider first the networks $N_s$, $N_w$, $N_n$ on 5 taxa of Fig. 9, called good ($N_w$) and bad ($N_s,N_n$) diamonds in Solís-Lemus and Ané (2016). Note that hybrid edge probabilities are not labeled for the networks $N_w$ and $N_n$, since no coalescence can occur in those edges as they have only one descendant taxon.

For any network *N*, an ideal ${\mathcal {J}}(N)$ of linear invariants is easy to construct from certain symmetries in *CF*s under the NMSC. There are, for example, *trivial invariants* like $1 - \left( CF_{ab|cd} + CF_{ac|bd} + CF_{ad|bc} \right)$, as well as *cut invariants* derived from cut edges in *N* and *exchange invariants* derived from exchangeable lineages under the NMSC. These linear invariants form a subideal ${\mathcal {J}}(N)$ of the full ideal ${\mathcal {I}} (N)$ for the network variety, and depend only on *N*’s undirected topology. See Appendix B.1 for full details.

Since $N_s$, $N_w$ and $N_n$ all have the same undirected topology,

$$\begin{aligned} {\mathcal {J}}(N_s)={\mathcal {J}}(N_w)={\mathcal {J}}(N_n), \end{aligned}$$

and the location of the hybrid node can not be determined using these linear invariants. However, computations of the full ideals of invariants for these three networks, presented as Propositions 38–40, with additional computation, yield the following identifiability result for hybrid nodes.

#### Proposition 18

Consider a semidirected binary level-1 network on $n\ge 5$ taxa whose topology is known up to contracting 2- and 3-cycles and undirecting hybrid edges in 4-cycles. Then for generic numerical parameter values on the network, the 4-cycle hybrid edge directions are identifiable from *CF*s.

#### Proof

**Fig. 10:**
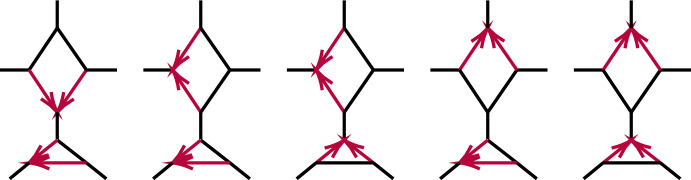
The semidirected 5-taxon level-1 binary networks with a single 4-cycle and 3-cycle, up to taxon labelling

Suppose first a network *N* has exactly 5 taxa, and a 4-cycle. Then after contracting 2-cycles *N* yields $N_s$, $N_w$, $N_n$, or one of the five networks shown in Fig. 10. Although we do not know whether *N* has a 3-cycle, if it does then by Proposition 6 it has the same associated variety as the network with that 3-cycle contracted, so we investigate the relationships of the varieties ${\mathcal {V}}(N_s)$, ${\mathcal {V}}(N_w),$ and ${\mathcal {V}}(N_n)$.

Proposition 38 to 40 show that ${\mathcal {V}}(N_s)$, ${\mathcal {V}}(N_w),$ and ${\mathcal {V}}(N_n)$ have dimensions 5, 4, and 3, respectively. Moreover, ${\mathcal {V}}(N_s)$ contains both ${\mathcal {V}}(N_w)$ and ${\mathcal {V}}(N_n)$. Additional computations show that the intersection

$$\begin{aligned} {\mathcal {V}}(N_w) \cap {\mathcal {V}}(N_n)= \mathcal {V}_1 \cup \mathcal {V}_2 \cup \mathcal {V}_3 \end{aligned}$$

has dimension 2, with three irreducible components ${\mathcal {V}}_1$, ${\mathcal {V}}_2$, ${\mathcal {V}}_3$ whose ideals are

$$\begin{aligned} \mathcal {I}(\mathcal {V}_1)&= \langle C\!F_{ab|cd} - C\!F_{ac|bd}, \, C\!F_{ab|ad} - C\!F_{ac|ad}, \, 3C\!F_{ac|ad}C\!F_{ac|bd} - C\!F_{ab|ac} \rangle ~+~ \mathcal {I}(N_s){,} \\ \mathcal {I}(\mathcal {V}_2)&= \langle C\!F_{ab|cd} + 2C\!F_{ac|bd} - 1, \, C\!F_{ab|ad} - C\!F_{ab|ac}, \,3C\!F_{ab|ac}C\!F_{ac|bd} - C\!F_{ac|ad} \rangle ~ +~ \mathcal {I}(N_s), \\ \mathcal {I}(\mathcal {V}_3)&= \langle C\!F_{ac|ad},\, C\!F_{ab|ac},\, C\!F_{ab|ad} \rangle ~+~ \mathcal {I}(N_s){.} \end{aligned}$$

Thus generic parameters for $N_s$ give points neither on ${\mathcal {V}}(N_w)$ nor ${\mathcal {V}}(N_n)$, while generic parameters for $N_w$ give points not on ${\mathcal {V}}(N_n)$, and generic parameters for $N_n$ give points not on ${\mathcal {V}}(N_w)$. Thus for generic parameters, the hybrid node in the 4-cycle can be determined by testing invariants to see whether the *CF*s lie on ${\mathcal {V}}(N_n)$ or ${\mathcal {V}}(N_w)$, or neither.

If *N* has more than 5 taxa, choose one taxon from each of 3 of the taxon blocks determined by a 4-cycle, and 2 from the remaining block, and pass to the induced network on these 5 taxa to apply the result for 5-taxon networks. $\square$

#### Remark 2

The components ${\mathcal {V}}_1, {\mathcal {V}}_2,$ and ${\mathcal {V}}_3$ of ${\mathcal {V}}(N_w) \cap {\mathcal {V}}(N_n)$ arise naturally from the parameterizations. Restricting to $\gamma =1$ on $N_w$ and $\gamma =0$ on $N_n$, essentially giving the unrooted tree $((a_1,a_2),(b,c),d)$ for both, yields ${\mathcal {V}}_1$. ${\mathcal {V}}_2$ arises from $\gamma =0$ on $N_w$ and $\gamma =1$ on $N_n$ which gives the unrooted tree $((a_1,a_2),b,(c,d))$. ${\mathcal {V}}_3$ arises from $\ell =0$ on both $N_w$ and $N_n$, which by corresponding to an infinite edge length, ensures $a_1,a_2$ form a cherry in any gene tree involving those two taxa, and for those involving only one $a_i$, gives *CF*s from a $4_1$-cycle with a non-identifable hybrid node.

### Summary of Topological Identifiability

The results of this section combined with Theorem 2 and Lemma 3 yield the following theorem.

#### Theorem 19

(Topological Identifiability from quartet *CF*s) Let $N^+$ be a binary level-1 phylogenetic network on $n \ge 4$ taxa, with generic numerical parameters. Then no 2-cycle on the semidirected network can be identified from *CF*s, so let ${\widetilde{N}}$ be the topological semidirected network induced by $N^+$ with all 2-cycles replaced with edges. Then the topological structure of ${\widetilde{N}}$, including directions of hybrid edges, is identifiable from quartet *CF*s of $N^+$, with the following exceptions:

1. If a 3-cycle induces a $(1,1,n-2)$ partition of taxa, then if the hybrid node has a single descendant taxon the network cannot be distinguished from the network in which the cycle is contracted to a node, or from the network in which the hybrid and other singleton block are interchanged. If the hybrid node has $n-2$ descendant taxa, then there are positive-measure subsets of parameters on which the semidirected 3-cycle is and is not identifiable.
2. If a 3-cycle induces a $(1,n_1,n_2)$ partition with $n_1,n_2\ge 2$ then the undirected 3-cycle can be identified. There are positive measure subsets of parameters on which the semidirected 3-cycle is and is not identifiable.
3. If a 3-cycle induces an $(n_1,n_2,n_3)$ partition with all $n_i\ge 2$, then the undirected 3-cycle can be identified, and at least 1 of the 3-cycle nodes can be determined not to be hybrid, but there are positive measure subsets of parameters on which the semidirected 3-cycle is and is not identifiable.
4. If a 4-cycle induces a (1, 1, 1, 1) partition, then the location of the hybrid node is not identifiable.

## Identifiability of Numerical Parameters

To address identifiability of numerical parameters—both edge lengths and hybridization parameters—we assume the network has no 2-cycles, as these are not identifiable. For the remainder of the section we thus study ${\widetilde{N}}$, the semidirected metric binary phylogenetic network induced from a rooted network $N^+$, with 2-cycles replaced by edges. In showing an edge in ${\widetilde{N}}$ has identifiable length, we are showing that if the original network did have a 2-cycle, then an “effective" length of an edge resulting from replacing the cycle as in Lemma 4 is identifiable.

Since we assume exactly one sample per taxon for each gene, no coalescent event can occur in pendant edges. Thus no pendant edge length appears in *CF* parameterizations, and such lengths cannot be identified from *CF*s, yielding the following.

### Proposition 20

Let *N* be a semidirected phylogenetic network. Then pendant edge lengths are not identifiable from quartet *CF*s under the NMSC model with one sample per taxon.

### Lengths of Edges Defined by 4 Taxa

We focus first on edges in ${\widetilde{N}}$ for which it is simple to identify edge lengths.

With $Q = \{a,b,c,d\}$ a set of 4 taxa from *X*, let ${\widetilde{N}}(Q)$ denote the subgraph of ${\widetilde{N}}$ obtained as is the induced quartet graph $N|_Q$ in Definition 1 but *without* suppressing degree-2 nodes.

#### Definition 4

Let *e* be an edge in ${\widetilde{N}}$. If $Q=\{a,b,c,d\}$ is a set of 4 taxa and ${\widetilde{N}}(Q)$ the subgraph of ${\widetilde{N}}$ described above, then we say that *e* is *defined* by a set *Q* if:

1. Edge *e* lies in the subnetwork ${\widetilde{N}}(Q)$,
2. Edge *e* is a cut edge of ${\widetilde{N}}(Q)$ separating pairs of taxa, say *a*, *b* from *c*, *d*, and
3. In ${\widetilde{N}}(Q)$ there are 4 cut edges adjacent to *e*, separating each of *a*, *b*, *c*, *d*, respectively, from the others.

**Fig. 11:**
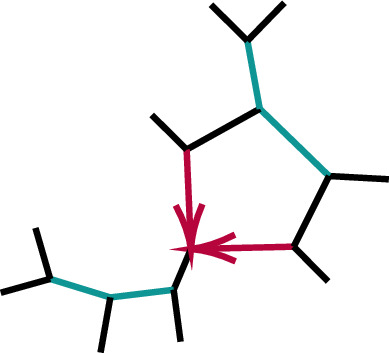
A semidirected network with edges defined by sets *Q* of 4 taxa highlighted in blue (Color figure online)

In an unrooted tree, every internal edge is defined by some *Q*, even if the tree is not binary. But for a network, even if binary and level-1 as in Fig. 11, this is not the case. In such a network, a *k*-cycle, with $k\ge 5,$ has $k-4$ edges in it that are defined by such sets, with the hybrid edges and those adjacent to them exceptions, as will be proved in the next proposition. Edges descended from hybrid nodes are also never defined by a set *Q*. These examples show edges defined by a set *Q* need not be cut edges, and not all cut edges are defined by a set *Q*.

For a binary network, an alternate characterization of edges defined by sets *Q* can be given.

#### Proposition 21

For a binary level-1 semidirected network ${\widetilde{N}}$, there is a set *Q* of 4 taxa defining an edge *e* if, and only if, *e* is an internal edge that is neither hybrid nor adjacent to a hybrid edge.

#### Proof

Suppose *e* is defined by *Q*. If *e* were either hybrid or adjacent to a hybrid edge, then *Q* would contain a descendant of a hybrid node. But then ${\widetilde{N}}(Q)$ contains all edges of the cycle in which the hybrid edge lies. This contradicts that both *e* and its adjacent edges are cut edges in ${\widetilde{N}}(Q)$, since the hybrid edges are not cut.

Conversely, suppose *e* is neither hybrid nor adjacent to a hybrid edge. If none of these 5 edges is in a cycle in ${\widetilde{N}}$, then choosing one taxon in each component obtained by deleting *e* and its incident nodes and adjacent edges gives a set *Q* defining *e*.

If any one of these edges is in a cycle, then since ${\widetilde{N}}$ is level-1 and binary, exactly one of the following holds: a) *e* is in a cycle, together with exactly 2 adjacent edges, one at each endpoint of *e*, b) *e* is not in a cycle, but exactly one cycle contains two edges adjacent to *e* at the same endpoint of *e*, or c) *e* is not in a cycle, but all 4 edges adjacent to *e* are, with *e* adjacent to two different cycles.

For case (a), the 2 edges adjacent to *e* that are not in the cycle must be cut edges, and the two adjacent to *e* that are in the cycle must be adjacent to 2 other distinct cut edges not in the cycle. Choosing taxa from the non-*e* components left by deleting these 4 cut edges gives a set *Q* defining *e*.

In case (b), The two edges in the cycle must be adjacent to distinct cut edges other than *e* which are not in the cycle. Choosing taxa from the non-*e* components of the graph obtained by deleting these two edges and the two non-cycle edges adjacent to *e* gives a quartet defining *e*. Case (c) is similar, treating each cycle the same way. $\square$

For any network, regardless of level or other special structure, lengths of edges defined by sets *Q* are easily identified.

#### Proposition 22

If an edge *e* in a metric network ${\widetilde{N}}$ is defined by a set *Q* of 4 taxa, then its length is identifiable from quartet *CF*s.

#### Proof

If *e* is defined by $Q=\{a,b,c,d\}$ has length *t* and in ${\widetilde{N}}|_Q$ induces the split *ab*|*cd*, then $\overline{CF}_{ac|bd}= \exp (-t)/3,$ so $t=-\log (3 CF_{ac|bd})$. $\square$

### Numerical Parameters Associated to 3-Cycles

Edges either in or adjacent to a 3-cycle are always adjacent to a hybrid edge. Thus in binary networks, these edges are not defined by sets of 4 taxa, so Proposition 22 does not apply. Propositions 33(c), 34(c) and 36(c) illustrate that, at least for specific small networks, the numerical parameters associated to 3-cycles are not identifiable. More generally, we obtain the following.

#### Proposition 23

If *C* is a 3-cycle on a semidirected binary level-1 network ${\widetilde{N}}$, then neither the hybridization parameters nor the lengths of any edges in or adjacent to *C* can be identified from quartet *CF*s.

#### Proof

Suppose first the 3-cycle induces an $(n_1,n_2,n_3)$-partition of the taxa with all $n_i\ge 2$. Then using Propositions 12 and 37(a) we see that the map from numerical parameters to *CF*s factors by sending the 7 numerical parameters associated to the 3-cycle and its adjacent edges into a 6-dimensional variety. This implies that the numerical parameters cannot all be identifiable. To see that no single parameter can be identified, first observe that from the factorization of maps in Eq. (5), if a single parameter were identifiable, it would have to be identifiable from a point in ${\mathcal {V}}_D$. However, Proposition 37(b) shows that is not the case.

If a 3-cycle induces a $(1,n_2,n_3)$- or $(1,1,n_3)$-partition of taxa, then by considering samples of 2 individuals for each gene from the singleton taxa, we can modify the network by attaching cherries of pseudotaxa for each singleton. Since in this case we already know that numerical parameters around the 3-cycle are not identifiable from all *CF*s, with access only to *CF*s using only one of the pseudotaxa, they are still not identifiable. But that means they are not identifiable for the original network. $\square$

### Other Numerical Parameters

The remaining numerical parameters on a binary level-1 network to be considered include lengths of hybrid edges, lengths of edges adjacent to hybrid edges, and hybridization parameters, all when the relevant cycle is of size $\ge 4$.

#### Proposition 24

Let ${\widetilde{N}}$ be a level-1 metric binary semidirected network with no 2-cycles, containing a *k*-cycle *C* with $k\ge 5$. Then hybridization parameters and lengths of the cycle edges adjacent to the hybrid edges in *C* can be identified from quartet *CF*s. If the hybrid node of *C* has at least 2 descendant taxa, the lengths of the hybrid edges can also be identified. If the hybrid node has only one descendant taxon then the lengths of the hybrid edges are not identifiable.

#### Proof

**Fig. 12:**
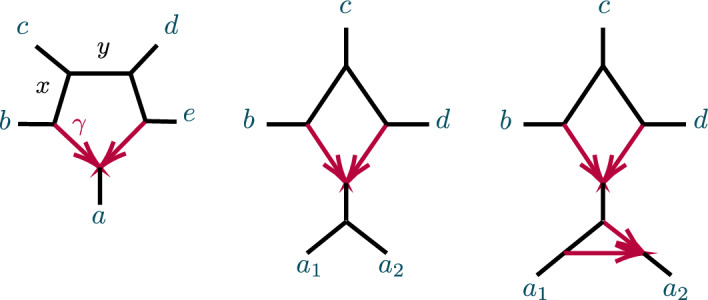
Subnetworks used in the proof of Proposition 24

From Proposition 22 we already know that the $k-4$ edges in the cycle that are not hybrid or adjacent to a hybrid edge have identifiable lengths. If the taxon blocks for the cycle are, proceeding from the hybrid around the cycle, $X_1,X_2,\ldots , X_k$, then pick one taxon from each of $X_1,X_2,X_3,X_4,$ and $X_k$ and pass to the induced subnetwork. Replacing any 2-cycles with edges, we may assume we have a 5-cycle sunlet network as in Fig. 12(left), in which the edge probability *y* of the edge opposite the hybrid node is known, and the edge probability *x* is that of the edge in *C* which is adjacent to a hybrid edge, lying between blocks $X_2$ and $X_3$.

Using *y* and *CF*s we can identify $\gamma$, and then *x* through

$$\begin{aligned} CF_{ac|de}-CF_{ad|ce}= \gamma \left( 1- y\right) ,\quad CF_{ab|cd}-CF_{ac|bd}= \gamma \left( 1- x\right) . \end{aligned}$$

Similarly, the other edge in *C* adjacent to a hybrid edge has identifiable length.

If the hybrid node has 2 descendant taxa, then by picking two taxa from $X_1$ and one from each of $X_2,X_3,X_k$ we pass to an induced subnetwork which, after replacing 2-cycles by edges, has the form of the network of Fig. 12(center) or (right) with the same hybrid edge lengths as the full network. In case (center), with a cherry below the hybrid node, applying the result of Proposition 38 (c) on $N_S$ identifies the hybrid edge lengths from *CF*s using the already identified $\gamma$. In case (right), a $3_1$-cycle below the hybrid node, by Proposition 6 all *CF*s are unchanged if the 3-cycle is contracted to a node and the edge length above it modified appropriately. Then the identifiability of the hybrid edge lengths follows from the cherry case.

If the hybrid node has only 1 descendant taxon, then at most 1 lineage may enter (going backwards in time) the hybrid edges of *C*, so no coalescent events may occur on the hybrid edges. Thus the *CF*s do not depend on the lengths of those edges, which are therefore not identifiable from *CF*s. $\square$

We next turn to cut edges adjacent to a single hybrid edge.

#### Proposition 25

Let ${\widetilde{N}}$ be a level-1 metric binary semidirected network with no 2-cycles, containing an internal cut edge *e*adjacent to exactly one hybrid edge (at its non-hybrid node), with the hybrid edge in a *k*-cycle. If $k\ge 4$, then the length of *e* is identifiable.

#### Proof

If $k\ge 4$, by passing to the induced network on a subset of the taxa, we may assume $k=4$. Since *e* is not pendant, and not adjacent to a hybrid edge of another cycle, after again passing to an induced subnetwork and replacing any 2-cycles with single edges, we may assume the network has the structure of $N_w$ in Fig. 9 (center), with *e* the edge joining the cherry to the 4-cycle. But then Proposition 39(c) gives the claim. $\square$

**Fig. 13:**
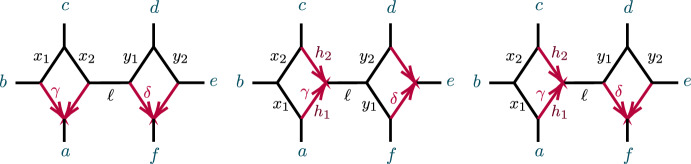
Semidirected binary networks on 6 taxa with two 4-cycles joined by an edge adjacent to two or more hybrid edges

If an edge is adjacent to hybrid edges at both of its endpoints, but neither endpoint is a hybrid node, as in Fig. 13 (left), then the following applies.

#### Proposition 26

Let ${\widetilde{N}}$ be a level-1 metric binary semidirected network with no 2-cycles, containing an edge $\epsilon$ adjacent to exactly two hybrid edges which lie in two different cycles. If the sizes of both cycles are $\ge 4$, then the length of $\epsilon$ is identifiable.

#### Proof

If both cycles are of size $\ge 4$, then the network has an induced subnetwork which, after suppressing 2-cycles has the form shown in Fig. 13 (left), with the central edge arising from $\epsilon$, with edge probability $\ell$.

Using Proposition 39 on the induced network after dropping taxon *f* we may identify $\gamma , x_1,x_2$ and the product $\ell y_1$. Similarly, dropping *a* we may identify $y_1$, which then gives $\ell$. $\square$

Next we consider edges adjacent to two hybrid edges at one endpoint, that is, edges with a hybrid node as an endpoint, as in Fig. 13 (center, right). If the hybrid node is in a large cycle we obtain the following.

#### Proposition 27

Let ${\widetilde{N}}$ be a level-1 metric binary semidirected network with no 2-cycles, containing an edge *q* whose parent is the hybrid node of a *k*-cycle with $k\ge 5$. If *q* has at least two descendant taxa, and the child node of *q* is not in a 3-cycle, then the length of *q* is identifiable.

#### Proof

Since the cycle is of size $\ge 5$, by Proposition 24 its hybridization parameter $\gamma$ is identified.

First suppose the child node of *q* is not incident to a hybrid edge. If *q* has two descendant taxa, there is an induced subnetwork which, after replacing 2-cycles by edges, has the form of $N_s$ of Fig. 9 (left), with *q* the child edge of the hybrid node. With $\gamma$ in hand, by Proposition 38(c) the length of *q* is identified.

If instead the child node of *q* is incident to a hybrid edge, assume that edge lies in a cycle of size $\ge 4$. We may then pass to a network with the structure of Fig. 13 (right) where *q* is the edge joining the two cycles. But dropping taxon *f* again yields a network of form $N_s$, so using $\gamma$ we identify $\ell y_1$. Instead dropping *b* from Fig. 13 (right), by Proposition 6, the 3-cycle on this can then be contracted to a node, adjusting the edge length of *q* (now possibly negative) so *CF*s are unchanged. Then Proposition 39 can be applied to identify $y_1$. Thus $\ell$ is identifiable. $\square$

The remaining parameters to consider are the edge probabilities and hybridization parameter in 4-cycles, and the edge probability of the child edge of the hybrid node in a 4-cycle. Identifiability of these is more complicated, as it can depend on the sizes of the taxon blocks of the cycle. In handling these cases, we use the following.

**Fig. 14:**
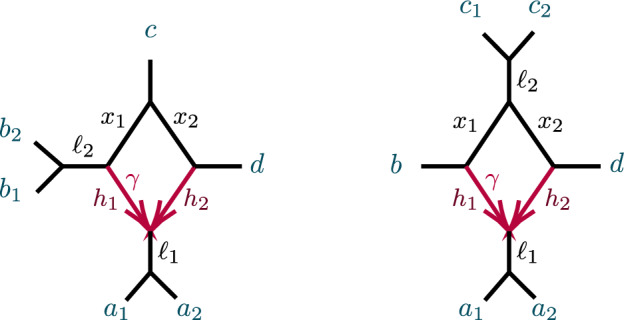
Semidirected binary networks on 6 taxa with a 4-cycle and two cherries: (left) $N_{sw}$ and (right) $N_{sn}$

#### Lemma 28

Consider a 6-taxon semidirected network with a 4-cycle, a cherry below the cycle’s hybrid node, and one other cherry, as shown in Fig. 14. Then all numerical parameters are identifiable from quartet *CF*s.

#### Proof

Consider Fig. 14 (left), $N_{sw}$. Then the subnetwork obtained by dropping taxon $a_2$ has the form of $N_w$, and Proposition 39 shows $\gamma ,x_1,x_2,\ell _2$ are identifiable. But the network obtained by dropping taxon $b_2$ has the form of $N_s$, so using Proposition 38 and the known value of $\gamma$ identifies $h_1,h_2,\ell _1$.

The identifiability of all parameters for Fig. 14(right), $N_{sn}$, follows from another computation, presented as Proposition 41. $\square$

**Fig. 15:**
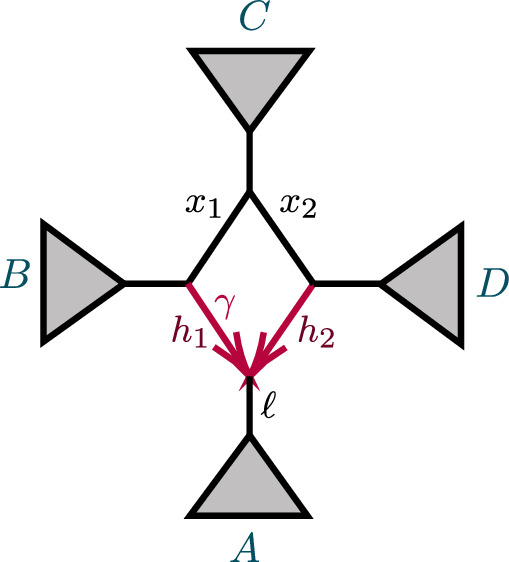
A 4-cycle in a larger network, partitioning the taxa into 4 blocks *A*, *B*, *C*, *D*

#### Proposition 29

Let ${\widetilde{N}}$ be a level-1 metric binary semidirected network on $n \ge 4$ taxa with no 2-cycles, containing a 4-cycle, as shown in Fig. 15, with taxon blocks *A*, *B*, *C*, *D* of size $n_A$, $n_B$, $n_C$, $n_D$ and edge probabilities and hybridization parameters on and below the cycle as shown. Then the parameters $x_1,x_2,h_1,h_2,\gamma , \ell$ are identifiable according to the following cases, at least one of which must hold.

- $$n_B=n_C=n_D=1$$: none identifiable
- $$n_A=1$$ and
  (i) $$n_B=n_D=1$$: none identifiable
  (ii) $n_B$ or $n_D\ge 2$: $x_1,x_2,\gamma$ identifiable, $h_1,h_2,\ell$ not identifiable
- $$n_A\ge 2$$; $$n_B$$, $$n_C$$, or $$n_D\ge 2$$ and
  (i) the child of the edge with probability $\ell$ is not in a 3-cycle: all identifiable
  (ii) the child of the edge with probability $\ell$ is in a 3-cycle: $x_1,x_2,h_1,h_2,\gamma$ identifiable, $\ell$ not identifiable

Simple instances of the 5 cases in the proposition may be helpful to consider. The network $N_s$ falls under case a), $N_n$ under b)i), $N_w$ under b)ii), and $N_{sw}$ and $N_{sn}$ under c)i). Examples for case c)ii) are obtained from $N_{sw}$ and $N_{sn}$ by replacing the cherry below the hybrid edge with a 3-cycle. The proof of the proposition leverages computational results for these to obtain more general statements.

#### Proof

That at least one of these cases must hold is most easily seen by noting that case c) is the complement of the union of a) and b). We consider each case to establish its claim.

**Case a):** The 4-cycle determines a hybrid block of taxa *A* and three taxa, *b*, *c*, *d*, in singleton blocks. If $n_A=1$, then the result is Lemma 3. If $n_A\ge 2$, the only *CF*s dependent on the parameters $\theta =(x_1,x_2,h_1,h_2,\gamma , \ell )$ are those involving at most two elements of *A*, since with 3 or 4 elements of *A* either a coalescence has occurred below the hybrid node, or at least 3 lineages reach it and are then exchangeable, giving probabilities 1/3 for each quartet tree. Those *CF*s dependent on $\theta$ decompose into sums of products of expressions involving only parameters outside of $\theta$ or only parameters in $\theta$, similar to the approach in Sect. 4.2.4. The expressions involving only parameters in $\theta$ can even be chosen from the *CF*s for the network $N_s$ of Proposition 38. But that Proposition shows the parameters in $\theta$ are not identifiable from the *CF*s for $N_s$, so they cannot be identified from those for ${\widetilde{N}}$.

**Case b)i):** The 4-cycle determines a hybrid singleton *a*, two adjacent singleton blocks of *b* and *d*, and a larger subnetwork *C* opposite the hybrid. Viewing the network as rooted in *C*, the *CF*s for ${\widetilde{N}}$ depend on parameters $x_1,x_2,h_1,h_2,\gamma , \ell$ only through the various probabilities of first coalescent events among subsets of $\{a,b,d\}$ determining the quartet tree before lineages leave the 4-cycle and enter *C*. Using ${\mathcal {D}}$ to denote the subnetwork below *C* which contains the 4-cycle, these are

$$\begin{aligned} p_1=P({{\mathcal {C}}_{{\mathcal {D}}}}\rightarrow ab|cc)&=\gamma (1-x_1){,} \\ p_2=P({{\mathcal {C}}_{{\mathcal {D}}}}\rightarrow ad|cc)&= (1-\gamma )(1-x_2){,} \\ P({{\mathcal {C}}_{{\mathcal {D}}}}\rightarrow bd|cc)&= 0{,} \\ P({{\mathcal {C}}_{{\mathcal {D}}}}\rightarrow bd|ac)&= (\gamma x_1 +(1-\gamma )x_2)/3=(1-p_1-p_2)/3{,} \\ P({{\mathcal {C}}_{{\mathcal {D}}}}\rightarrow ab|dc)&= \gamma \left( 1- 2 x_1/3\right) +(1-\gamma ) x_2/3=(1+2p_1-p_2)/3{,} \\ P({{\mathcal {C}}_{{\mathcal {D}}}}\rightarrow ad|bc)&= \gamma x_1/3+(1-\gamma )\left( 1-2 x_2/3\right) =(1-p_1+2p_2)/3{.} \end{aligned}$$

Since these probabilities are linear functions of $p_1,p_2$, and none of $\gamma ,x_1,x_2$ are identifiable from $p_1,p_2$, none of the parameters are identifiable from *CF*s for ${\widetilde{N}}$.

**Case b)ii):** Pick two taxa in one of the blocks adjacent to the hybrid one, and one taxon in all others. Passing to the induced subnetwork and removing 2-cycles yields either a network with the form $N_w$ or one where the cherry in $N_w$ is replaced by a 3-cycle. Using Proposition 6, we may replace such a 3-cycle with a node without changing *CF*s, provided we modify the edge length leading to the 4-cycle, including allowing for a possibly negative branch length. But then the network has the form $N_w$ and applying Proposition 39 shows $\gamma , x_1,x_2$ can be identified.

Since there is only one taxon descended from the hybrid node, there can be no coalescent event in either of the hybrid edges or their descendant, and thus these edge lengths do not appear in the formulas for the *CF*s for ${\widetilde{N}}$. Therefore these parameters cannot be identifiable.

**Case c)i):** Pick two taxa in one of the non-hybrid blocks, two taxa in the hybrid block, and one taxon from each of the others. Passing to the induced subnetwork on these 6 taxa, and removing any 2-cycles, we obtain a network of one of the forms in Fig. 14, or ones where 3-cycles appear in place of one or both cherry nodes. If there are 3-cycles, by Proposition 6 we may replace them with nodes without changing *CF*s (provided we modify edge lengths leading to the 4-cycle). Then using Lemma 28 we can identify $\gamma ,x_1,x_2,h_1,h_2$.

To identify $\ell$, let *v* be the child node of the edge with this probability. If *v* is not in a cycle in ${\widetilde{N}}$, then picking one taxon descended from each of its child edges and passing to an induced subnetwork, $\ell$ is identifiable by Lemma 28.

If *v* is in a cycle, it is of size $\ge 4$. Passing to an induced subnetwork, we may assume that *v* is in a 4-cycle. Note that *v* cannot be the hybrid node of that cycle, else the semidirected network would not be rootable. If *v* is opposite the hybrid node, then we may pass to an induced subnetwork which, after replacing 2-cycles with edges, has a cherry below *v* and follow the previous argument. If *v* is adjacent to the hybrid node, then the subnetwork has the form of Fig. 13(right). Since $\gamma$ is identified, the argument used in Proposition 27 then shows $\ell$ is identifiable.

**Case c)ii):** The argument of the first paragraph for Case c)i) shows $\gamma ,x_1,x_2,h_1,h_2$ are identifiable. Since the edge descending from the hybrid node of the 4-cycle is incident to a 3-cycle, its length is not identifiable by Proposition 23. $\square$

### Summary of Numerical Parameter Identifiability

We summarize this section’s results with the following.

#### Theorem 30

(Numerical parameter identifiability from quartet *CF*s) Let ${\widetilde{N}}$ be a level-1 metric binary semidirected network on *X* with no 2-cycles, and $\vert X \vert \ge 4$. Then from quartet *CF*s under the NMSC with one sample per taxon all numerical parameters on ${\widetilde{N}}$ are identifiable except for the following, which are not identifiable:

1. Pendant edge lengths,
2. For 3-cycles, hybridization parameters and the lengths of the six edges in and adjacent to the cycle,
3. For 4-cycles, the hybridization parameter and edge lengths in the cycle and descended from the hybrid node, as stated in Proposition 29.

If two individuals are sampled in some taxon *x*, as discussed earlier this can be modeled by attaching a cherry of pseudotaxa $x_1,x_2$ at the leaf *x*. Doing so for all taxa resolves the non-identifiability issues of Items 1 and 3, yielding the following.

#### Corollary 31

Let ${\widetilde{N}}$ be a level-1 metric binary semidirected network with no 2-cycles. Then from quartet *CF*s under the NMSC with two or more samples for all taxa, all numerical parameters on ${\widetilde{N}}$ are identifiable except hybridization parameters and lengths of edges in and adjacent to 3-cycles.

## Implications for Data Analysis

Attempting to infer the non-identifiable can either be misleading (unless all possible alternatives are reported), or very slow (spending computational time considering equally good possibilities), so our results here should inform development and use of *CF*-based inference methods.

The issues with identifiability of 3-cycles from *CF*s under the NMSC shown here are perhaps the greatest source of problems for practical inference. Hybridization or gene flow is generally believed to occur most frequently among recently diverged populations, and when this occurs between sister populations it leads to a 3-cycle. Thus these cycles may commonly underlie empirical data. We have shown that in many cases *CF*s may indicate the presence of a 3-cycle, though not necessarily its hybrid node, but that the numerical parameters associated to it are not identifiable.

This poses particular issues for likelihood and pseudolikelihood approaches. Quartet *CF*s may carry signals of undirected 3-cycles (even in certain “bad triangle” cases not considered in SNaQ’s search), and ignoring the possibility of such cycles could have unknown consequences under these optimality criteria. Since for some parameter values there is a signal of a 3-cycle’s hybrid node in the *CF*s, the search cannot be limited to undirected 3-cycles in all circumstances.

Even if only the network topology is sought, these criteria require optimization over numerical parameters, so these must be dealt with in a search. However, since the numerical parameters are not identifiable, searching over them directly will be slow. Reducing the over-parameterization at 3-cycles (e.g., from 7 parameters to 6 when the 3-cycle is not near a leaf) is desirable, but how to do so while maintaining the same range of *CF*s is unclear. Even if this were accomplished, as the numerical parameters vary, the semidirected topology may pass between identifiable and non-identifiable regimes, and the boundaries of these are not known. Without such information, one must consider all possibilities for the location of a hybrid node in a 3-cycle throughout, but allow for multiple optimal networks.

SNaQ (Solís-Lemus and Ané 2016), with its default settings, restricts its search for 3-cycles in networks to those with all cycle blocks of size at least 2 (“good triangles”), corresponding to our Theorem 19 (3). It addresses the numerical overparameterization at 3-cycles by setting the edge probability below the putative hybrid node ($\ell _1$ in Fig. 6, right) to 1, reducing the number of numerical parameters to be estimated to six ($\gamma , h_1, h_2, x, \ell _2, \ell _3$), matching the dimension of the variety. The estimated parameters are then *composite* parameters, which are functions of the original ones producing the same *CF*s. While our computations (not shown) indicate that this parameterization of *CF*s is 2-to-1, unlike the 1-to-1 map suggested by Proposition 37, that is not necessarily problematic but could result in multiple optima.

More worrisome is the fact that both of these maps are only guaranteed to give *complex* parameterizations of the relevant variety, and when restricted to stochastic parameters do not necessarily produce the same collection of *CF*s as the original map. We experimented with 40,000 sets of 7 parameters ($\gamma$ and 6 edge probabilities) chosen uniformly-at-random in [0, 1] for the network in Fig. 6(right), and found in 93.4% of the cases there were no stochastic parameters with $\ell _1=1$ producing the same *CFs*. In 6.4% there was a single stochastic parameter choice producing the *CF*s, and in the remaining 0.2% there were two. A full numerical parameter search with the SNaQ approach thus requires examining non-stochastic parameter values (negative, $> 1$, or even complex), and then verifying optimal values give *CF*s that also arise from some set of stochastic parameters (without $\ell _1$ restricted to 1). While limiting the search space to both be lower dimensional and only give *CF*s arising from stochastic parameters would be desirable, how to do so is an open problem.

Since exactly what information in *CF*s is extracted by maximizing the pseudolikelihood function is difficult to analyze theoretically, using simulation the impact of 3-cycles on inference needs to be studied thoroughly, both for SNaQ and for PhyloNet’s similar inference from rooted triples (Yu and Nakhleh 2015). NANUQ (Allman et al. 2019) does not suffer from these problems, as its inference goal is more modest, providing a statistically consistent estimate only of larger cycle topology, without any search over the numerical parameter space. Whether NANUQ can be supplemented to extract *CF* information on the existence of 3-cycles should also be explored.

Although our goal in this work was to understand the theoretical question of parameter identifiability from *CF*s under the NMSC for level-1 networks, some of our results for small networks also address the question of practical identifiability for networks with any number of taxa. For example, a true reticulate evolutionary history for a large number of taxa might be described by a graph containing a 4-cycle in which some or all of the cut edges leading to cycle blocks are long. Long branch lengths (in coalescent units) can arise from either small population sizes (bottlenecks) or long times in generations. Regardless, the probability that all lineages entering those long edges coalesce before entering the cycle may be close to 1, almost ensuring that only a single lineage reaches the 4-cycle from such a cut edge. This reduces what parameters may be practically identifiable from a finite data set, with the extreme case of a single lineage from each of the 4 cut edges yielding only the undirected topological 4-cycle, and no numerical information. Using standard likelihood-based approaches, network details such as these may be inferred and reported, even when there is little signal in the data supporting them.

In closing we remark that identifiability theorems needed to justify network inference methods from data types other than *CF*s are largely lacking. Studies of the parameter identifiability question for these data are urgently needed as well.

## Figures and Tables

**Fig. 16:**
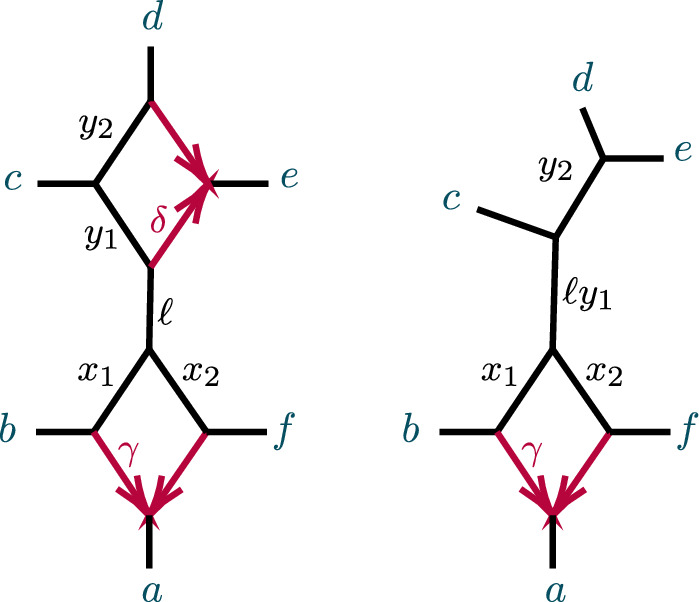
(left) A network with a 4-cycle of interest at bottom, and (right) a network with a single cycle obtained by removing a hybrid edge from each pair not in the cycle of interest

## Appendix A: Context for This Work

### A.1 Earlier Work

Here we discuss how results in this work fit into and complement earlier work. In what follows, all statements should be restricted to the class of level-1 binary networks.

Questions of identifiability of both network topology and numerical parameters from quartet *CF*s were first considered in Solís-Lemus and Ané (2016), a work which also introduced the pseudolikelihood inference of networks from empirical *CF*s via the SNaQ software. With the publication directed primarily to a biological audience, much of the presentation of the mathematical results appeared in the supplementary material, without formal statements of propositions and theorems, making it difficult to discern exactly what has been fully established. Subsequently, Baños (2019) reconsidered the identifiability of level-1 network topology from quartet *CF*s with more mathematical formality. A later preprint (Solis-Lemus et al. 2020) gives more details on the work behind Solís-Lemus and Ané (2016) and its supplement.

As does this paper, Solís-Lemus and Ané (2016) uses algebraic computations to study what information quartet *CF*s might carry about a network. However, the computations concerning network topology are focused not on the full question of identifiability, but on a more restricted question the authors called *detectability* of hybridizations. This notion is an instance of the broader concept of *distinguishability* presented subsequently in Degnan (2018). Distinguishability for a specified collection of possible models using some specified information source means that a model *x* that is known to be in the collection can be determined from that information for *x*. In Solís-Lemus and Ané (2016) the models correspond to certain networks, and the information is expected *CF*s arising from them under the NMSC. As stated in Solís-Lemus and Ané (2016), ...for networks with $n\ge 4$ taxa, we restrict our focus to the case when $N'$ is the network topology obtained from *N* by removing a single hybrid edge of interest. ...The presence of the hybridization of interest can be detected if the quartet *CF*s from $N'$ cannot all be equal to the quartet *CF*s from *N* simultaneously. In other words, that work focused on distinguishability of the 2-element set $\{N,N'\}$ using quartet *CF*s. While investigating this question was certainly a strong first contribution to the broader question of identifiability of level-1 network topology from *CF*s, addressing the full question would require showing distinguishability of the set of *all* possible level-1 networks on a taxon set *X*, including networks with completely unrelated topologies. More fully, one would need to show this for sets *X* of arbitrary size. The approach taken to prove detectability by Solís-Lemus and Ané (2016), however, depends on equating formulas for *CF*s in terms of numerical parameters on the two networks and solving the system, and it is unclear how this could be applied to the full identifiability question.

Note that while Solís-Lemus and Ané (2016) states that their detectability results extend to level-1 networks with many cycles (where multiple hybrid edges may be removed to get the set of distinguishable networks), a justification for this claim was only given in Solis-Lemus et al. (2020), with Lemma 3 of that work being key. Unfortunately, that lemma is incorrect as stated. (See Sect. A.2 of this appendix for a counterexample.) While it might be possible to obtain a correct justification using similar ideas, doing so seems unnecessary given the results of Baños (2019), which we describe next.

After presenting a detailed study of all level-1 networks on 4 taxa and their *CF*s, Baños (2019) used combinatorial arguments to show that information about larger networks could be obtained through induced quartet networks, and hence *CF*s. In particular, the topological identifiability result stated in this paper as Theorem 2 was established. This work also clarified several points that were either implicit or unaddressed in Solís-Lemus and Ané (2016): First, the semidirected network was formally defined, highlighting that network structure above the LSA needed to be excised. Second, identifiability of large cycles (more than 4 edges) was explicitly addressed (although Solis-Lemus et al. 2020 later provided an argument for detectability). Third, identifiability for networks with multiple cycles was shown. However, because it considered only one *CF* at a time to deduce information about an induced quartet network, without exploring whether additional information might be found in the relationship of *CF*s for overlapping sets of taxa, it did not obtain the strongest possible result. As an example, while Solís-Lemus and Ané (2016) showed the distinguishability of the hybrid node of a 4-cycle in certain sufficiently large networks, results in (Baños 2019) simply left all 4-cycles undirected in its network identifiability result.

Moreover, in (Solís-Lemus and Ané 2016) it was shown that in some cases 3-cycles were detectable, while the main result of Baños (2019) omits 3-cycles in its identifiability result. It did contain a theorem, though, suggesting a $3_2$-cycle on a 4-taxon network may be identifiable for some parameter values. In both works, then, questions about 3-cycle identifiability were left open.

In short, the general question, in arbitrary level-1 networks, of topological identifiability of both directed and undirected 3-cycles and of directed 4-cycles, the focus of Sects. 4.2 and 4.3 of this work, remained.

Although not considered by Baños (2019), the study of identifiability of numerical parameters was also initiated by Solís-Lemus and Ané (2016). Using calculations of Gröbner bases of ideals of polynomial relationships between *CF*s, arguments in Solís-Lemus and Ané (2016) (with a technical matter corrected in Solis-Lemus et al. 2020) investigated whether the dimension of the associated algebraic variety allowed for all numerical parameters to be identifiable. If this dimension is less than the number of parameters, then not all parameters can be identified, though it is possible that a subset are. If the dimension and number of parameters are equal, then the parameterization map must be generically finite-to-1. For specific networks Solís-Lemus and Ané (2016) determined whether or not the parameterization was finite-to-1, but passing to networks with more than 1 cycle again depended on the faulty Lemma 3 of Solis-Lemus et al. (2020). Moreover, the calculations in Solís-Lemus and Ané (2016) are focused on numerical parameters associated to cycles (cycle edge lengths, hybridization parameters, and possibly lengths of edges adjacent to a cycle). Although the arguments do not seem to cover edges not adjacent to any cycles, this omission is easily overcome (for instance as in our Proposition 22). On the other hand, it is unclear how the computations can be applied to answer whether the edges adjacent to hybrid edges in two different cycles have identifiable lengths. Moreover, when a dimension computation leads to a valid conclusion that a full set of numerical parameters cannot be identified, it still is possible that some subset of the parameters could be. This issue was not explored.

Finally, establishing that there can only be finitely many choices of parameters that yield the *CF*s on a network is a *local identifiability* result, and Solís-Lemus and Ané (2016) emphasized it leaves open the question of *global identifiability*: Does this finite set actually contain only one such choice? This cannot be settled by dimension computations of the sort in Solís-Lemus and Ané (2016). There are in fact simple statistical models (outside of phylogenetics) where parameterizations are finite-to-1, but not 1-to-1, in surprising ways (Allman et al. 2015). Investigating global identifiability is thus highly desirable.

Several questions of identifiability of numerical parameters questions thus remained open: identifiability of certain edge lengths, especially in multicycle networks; identifiability of subsets of parameters when a full set was not identifiable; and the broad question of global identifiability. These questions are the focus of Sect. 5.

When identifiability of individual parameters fails, it remains possible that composite parameters (i.e., functions of the parameters) might be identifiable and used in inference. For instance, for the network in Lemma 3, Solís-Lemus and Ané (2016) observed that $CF_{ab|cd} - CF_{ac|bd} = \gamma (1 - x_1)$ and $CF_{ad|bc} - CF_{ac|bd} = (1-\gamma )(1-x_2)$. In optimizing the pseudolikelihood function, SNaQ makes use of composite parameters in some situations when the original ones are not identifiable. While this can be an important issue in algorithm design, we do not focus on it in this work, though some of these known composite parameters appear in our arguments.

As the manuscript for this work was being completed, the preprint (Tiley and Solis-Lemus 2023) appeared. This includes work on distinguishability of several different 6-taxon networks with 4-cycles and several cherries, but not on the full network identifiability questions even for 6-taxon networks with 4-cycles. It does however include simulation work to investigate *practical identifiability*, the extent to which with finite data sets one can infer such cycles, using several pseudolikelihood methods.

### A.2 An Example

Consider the two networks shown in Fig. 16, where the right network is obtained from the left by removing a hybrid edge in the top 4-cycle. This is an instance of the construction implied in the statement of Lemma 3 of Solis-Lemus et al. (2020), which is used to justify analyzing *CF*s only of level-1 networks with a single cycle to understand those with multiple cycles. We show here that the statement of Lemma 3 is not correct for this pair of networks.

We first note that this lemma states that the *CF*’s for the two networks which depend on parameters associated to the cycle are equal. This is not strictly true as stated: Focusing on the lower cycle of the left network, for instance, $CF_{ab|ce}$ depends on $\gamma , x_1$ as well as on $\delta$ for the left network, but for the right network has no dependence on $\delta$. Other interpretations of this statement are that the described set of *CF*s for the two networks produce the same values as parameters vary, or that the varieties defined by the parameterized *CF*s are the same. As this last interpretation is the broadest, we show it is also false.

Consider $CF_{ac|df }$ and $CF_{ac|ef}$ for the two networks. For the left network

$$\begin{aligned} CF_{ac|df}&=\frac{1}{3} \ell y_1( \gamma +(1-\gamma )x_2),\\ CF_{ac|ef}&= \frac{1}{3} \ell ( \gamma +(1-\gamma )x_2)(\delta +(1-\delta )y_1), \end{aligned}$$

which are not equal for generic parameter values. However, for the right network

$$\begin{aligned} CF_{ac|df }= CF_{ac|ef} = \frac{1}{3} \ell y_1( \gamma +(1-\gamma )x_2) \end{aligned}$$

since *d*, *e* can be exchanged in the graph because they form a cherry. Thus the variety for the right network has a defining equation that does not hold for the left.

This example illustrates an important point that in passing to smaller induced networks a cycle other than the one of immediate focus may have an impact on *CF*s. In particular, a more detailed treatment of networks with multiple cycles, such as our work in Sect. 4.3, seems to be a necessary part of addressing the full identifiability question.

## Appendix B: Propositions from Computations

### B.1 Ideals of *CF* Invariants

Several observations simplify investigating the ideals ${\mathcal {I}}(N)$ described in Sect. 3. First, since the entries of any vector concordance factor add to 1, at most two of the 3 entries of a $\overline{CF}$ are linearly independent. We consider the polynomials expressing that $\overline{CF}$ entries add to 1 to be *trivial invariants*, as they hold for all network topologies. If only 4 taxa *a*, *b*, *c*, *d* are considered, then the trivial invariant is

$$\begin{aligned} CF_{ab|cd} +CF_{ac|bd} + CF_{ad|bc}-1, \end{aligned}$$

while for *n* taxa there are $n\atopwithdelims (){4}$ such invariants, one for each possible quartet.

Second, if there is a cut edge on an induced quartet network separating two of the taxa, *a*, *b* from the others *c*, *d*, then the *CF*s associated with the discordant topologies *ac*|*bd* and *ad*|*bc* must be equal. This was shown in the level-1 case in Baños (2019) and for general networks in Allman et al. (2022). The invariant expressing this is

$$\begin{aligned} CF_{ac|bd}-CF_{ad|bc}. \end{aligned}$$

We call such polynomials *cut invariants*.

Third, when a network has one or more cherries (2 taxa joined by pendant edges to a common node), we will label the taxa in each cherry by the same subscripted letter, such as $a_1,a_2$. (See for instance Fig. 5.) In *CF*s involving such taxa, we may then suppress the subscripts, since under the NMSC model on a suitably rooted version of the semidirected network the taxa $a_1$ and $a_2$ are exchangeable, giving the same *CF* values when they are interchanged. Thus, for example, on a network with a cherry formed by $a_1,a_2$,

$$\begin{aligned} CF_{ab|cd}=CF_{a _1b|cd}=CF_{a _2b|cd}, \end{aligned}$$

and

$$\begin{aligned} CF_{ab|ac}=CF_{a_1b|a_2c }=CF_{a_2b|a_1c}. \end{aligned}$$

We call these *exchange invariants*, but note that some of these these are also cut invariants. For computations, our notational simplification of surpressing subscripts allows for their omission.

#### Definition 5

For a topological phylogenetic network *N*,  the ideal generated by all trivial, cut, and exchange invariants of *N* is denoted ${\mathcal {J}}( N)$.

Note that ${\mathcal {J}}( N)$ depends on the topology of the network *N* due to the cut and exchange invariants. However, it only depends on the undirected topology. Also, while ${\mathcal {J}}( N)\subseteq {\mathcal {I}}(N)$ these ideals are typically not equal. Since the generators of ${\mathcal {J}}(N)$ are linear, and simple to enumerate, we use them for removing some *CF*s from calculations of ${\mathcal {I}}(N)$, and for stating results on ideal generators more succinctly.

### B.2 Propositions from 3-Cycle Computations

#### Proposition 32

Let $T_5=((a_1,a_2),c,(b_1,b_2))$ be a 5-taxon tree, as shown in Fig. 5 (left). Then

- The quartet concordance factors of $T_5$ are
  $$\begin{aligned} \overline{CF}_{aabc}&= \left( 1 -\frac{2}{3}\ell _1,\ \frac{1}{3}\ell _1,\ \frac{1}{3}\ell _1 \right) , \\ \overline{CF}_{acbb}&= \left( 1 -\frac{2}{3}\ell _2,\ \frac{1}{3}\ell _2,\ \frac{1}{3}\ell _2 \right) , \\ \overline{CF}_{aabb}&= \left( 1 -\frac{2}{3}\ell _2\ell _1,\ \frac{1}{3}\ell _2\ell _1,\ \frac{1}{3}\ell _2\ell _1 \right) . \end{aligned}$$
- The ideal defining $\mathcal {V}_{T_5}\subset \mathbb {C}^{9}$ is ${\mathcal {I}}(T_5) = {\mathcal {J}}(T_5) + \langle f_{abc} \rangle ,$ where
  $$\begin{aligned} f_{abc}= 3CF_{ab|ac}CF_{ba|bc} - CF_{ab|ab}. \end{aligned}$$
  The variety $\mathcal {V}_{T_5}$ has dimension 2.
- The numerical parameters $\ell _1$ and $\ell _2$ can be determined from quartet *CF*s:
  $$\begin{aligned} \ell _1 = 3CF_{ab|ac}, \quad \ell _2 = 3CF_{ab|cb}. \end{aligned}$$

#### Proposition 33

Let $N_{5-3_1}$ be a 5-taxon level-1 network with a central $3_1$-cycle, as in Fig. 5 (center). Then

- The quartet concordance factors of $N_{5-3_1}$ are
  $$\begin{aligned} \overline{CF}_{aabc}&= \left( 1 - \frac{2}{3}\ell _1(\gamma + x-\gamma x),\ \frac{1}{3}\ell _1(\gamma + x-\gamma x),\ \frac{1}{3}\ell _1(\gamma + x-\gamma x) \right) , \\ \overline{CF}_{acbb}&= \left( 1 - \frac{2}{3}\ell _2(1-\gamma + \gamma x),\ \frac{1}{3}\ell _2(1-\gamma + \gamma x),\ \frac{1}{3}\ell _2(1-\gamma + \gamma x) \right) , \\ \overline{CF}_{aabb}&= \left( 1 -\frac{2}{3}\ell _2\ell _1x,\ \frac{1}{3}\ell _2\ell _1x,\ \frac{1}{3}\ell _2\ell _1x \right) . \end{aligned}$$
- The ideal defining $\mathcal {V}({N_{5-3_1}})\subset \mathbb {C}^{9}$ is ${\mathcal {I}}(N_{5-3_1})={\mathcal {J}}(N_{5-3_1})={\mathcal {J}}(T_5)$, The variety $\mathcal {V}_{N_{5-3_1}}$ has dimension 3.
- None of the numerical parameters $\gamma$, *x*, $\ell _1$, $\ell _2$ can be determined from quartet *CF*s. They can be determined with one degree of freedom, for instance if $\ell _1$ is known:
  $$\begin{aligned} \begin{aligned} \ell _2&= \frac{3\ell _1CF_{ab|cb} - 3CF_{ab|ab}}{\ell _1 - 3CF_{ab|ac}}, \quad x = \frac{\ell _1CF_{ab|ab} - 3CF_{ab|ab}CF_{ab|ac}}{\ell _1^2CF_{ab|cb} - \ell _1CF_{ab|ab}}, \\ \gamma&= \frac{3\ell _1CF_{ab|cb}CF_{ab|ac} - \ell _1CF_{ab|ab}}{\ell _1^2CF_{ab|cb} - 2\ell _1CF_{ab|ab} + 3CF_{ab|ab}CF_{ab|ac}}. \end{aligned} \end{aligned}$$

#### Proposition 34

Let $N_{5-3_2}$ be a 5-taxon level-1 semidirected network with a central $3_2$-cycle, as in Fig. 5 (right). Then,

- The quartet concordance factors of $N_{5-3_2}$ are
  $$\begin{aligned} \overline{CF}_{aabc} =&\left( 1-\frac{2}{3}\ell _1u,\ \frac{1}{3}\ell _1u,\ \frac{1}{3}\ell _1u \right) , \text { with } u = \gamma ^2h_1 + \gamma (1-\gamma )(3-x)+ (1-\gamma )^2h_2, \\ \overline{CF}_{acbb} =&\left( 1-\frac{2}{3}\ell _2v,\ \frac{1}{3}\ell _2v,\ \frac{1}{3}\ell _2v \right) , \text { with } v=\gamma + (1-\gamma ) x ,\\ \overline{CF}_{aabb} =&\left( 1-\frac{2}{3}\ell _1\ell _2w,\ \frac{1}{3}\ell _1\ell _2w,\ \frac{1}{3}\ell _1\ell _2 w \right) , \text { with } w = \gamma ^2h_1 + 2\gamma (1-\gamma )+ (1-\gamma )^2 h_2x . \end{aligned}$$
- The ideal defining $\mathcal {V}_{N_{5-3_2}}\subset \mathbb {C}^{9}$ is ${\mathcal {I}}(N_{5-3_2})={\mathcal {J}}(N_{5-3_2})={\mathcal {J}}(T_5)$. The variety $\mathcal {V}_{N_{5-3_2}}$ has dimension 3.
- None of the numerical parameters $\gamma$, $h_1$, $h_2$, *x*, $\ell _1$, $\ell _2$ can be determined from quartet *CF*s. They can be determined with three degrees of freedom. If $\gamma$, $\ell _1$ and $\ell _2$ are known then:
  $$\begin{aligned} \begin{aligned} x&= \frac{\gamma \ell _2 - 3CF_{ab|bc}}{\gamma \ell _2 - \ell _2}, \quad h_2 = \frac{\gamma \ell _1\ell _2 - 3\gamma \ell _1CF_{ab|bc} - 3\ell _2CF_{ab|ac} + 3CF_{ab|ab}}{\ell _1\left( \ell _2 - 3CF_{ab|bc}\right) (\gamma - 1)}, \\ h_1&= \frac{1}{\gamma ^2\ell _1\ell _2\left( \ell _2-3CF_{ab|bc}\right) } \Big [ 3\left( \gamma \ell _1\left( -3CF_{ab|bc}-\gamma \ell _2+3\ell _2\right) -3\ell _2CF_{ab|ac}\right) CF_{ab|bc}\\&\quad + 3\gamma \ell _2^2CF_{ab|ac} + 3\ell _2CF_{ab|ab}(1-\gamma ) - \gamma \ell _1\ell _2^2(2-\gamma ) \Big ]. \end{aligned} \end{aligned}$$

#### Proposition 35

Let $T_6$ be the 6-taxon tree with a central node and 3 cherries, as in Fig. 6 (right). Then,

- The quartet concordance factors of $T_6$ are
  $$\begin{aligned} \overline{CF}_{aabb}&=\left( 1-\frac{2}{3}\ell _2\ell _1,\frac{1}{3}\ell _2\ell _1,\frac{1}{3}\ell _2\ell _1\right) , \\ \overline{CF}_{aabc}&=\left( 1- \frac{2}{3}\ell _1 , \frac{1}{3}\ell _1, \frac{1}{3}\ell _1\right) ,\\ \overline{CF}_{abbc}&=\left( \frac{1}{3}\ell _2,\frac{1}{3}\ell _2 ,1-\frac{2}{3}\ell _2\right) , \\ \overline{CF}_{abcc}&=\left( 1-\frac{2}{3}\ell _3 , \frac{1}{3}\ell _3,\frac{1}{3}\ell _3 \right) , \\ \overline{CF}_{aacc}&=\left( 1-\frac{2}{3}\ell _3\ell _1 ,\frac{1}{3}\ell _3\ell _1,\frac{1}{3}\ell _3\ell _1\right) , \\ \overline{CF}_{bbcc}&=\left( 1- \frac{2}{3}\ell _3\ell _2 , \frac{1}{3}\ell _3\ell _2, \frac{1}{3}\ell _3\ell _2 \right) . \end{aligned}$$
- The ideal defining $\mathcal {V}_{T_6}\subset {\mathbb {C}}^{18}$ is ${\mathcal {I}}(T_6) = {\mathcal {J}}(T_6) + \langle f_{abc}, f_{bca}, f_{cab}\rangle$ where
  $$\begin{aligned}&f_{abc} = 3CF_{ab|ac}CF_{ab|bc} - CF_{ab|ab},\\&f_{bca} = 3CF_{ab|bc}CF_{ac|bc} - CF_{bc|bc},\\&f_{cab} = 3CF_{ab|ac}CF_{ac|bc} - CF_{ac|ac}. \end{aligned}$$
  The variety $\mathcal {V}_{T_6}$ has dimension 3.
- The numerical parameters $\ell _1$, $\ell _2$, $\ell _3$ can be determined from quartet *CF*s by:
  $$\begin{aligned} \ell _1= 3CF_{ab|ac},\quad \ell _2 = 3CF_{ab|bc},\quad \ell _3 = 3CF_{ac|bc}. \end{aligned}$$

#### Proposition 36

Let $N_{6-3_2}=N_a$ be a 6-taxon level-1 semidirected network with a central $3_2$-cycle and 3 cherries, as shown in Fig. 6 (left). Then,

- The quartet concordance factors of $N_{6-3_2}$ are
  $$\begin{aligned} \overline{CF}_{aabb}&=( 1-2\alpha ,\alpha ,\alpha ),\quad \text {with}\quad \alpha = \frac{1}{3}\ell _1\ell _2(\gamma ^2h_1 +2\gamma (1-\gamma )+ (1-\gamma )^2h_2x ) ,\\ \overline{CF}_{aabc}&=(1-2\beta ,\beta ,\beta ), \quad \text {with}\quad \beta = \frac{1}{3}\ell _1(\gamma ^2h_1 + \gamma (1-\gamma )(3-x)+ (1-\gamma )^2h_2 ),\\ \overline{CF}_{abbc}&=({\eta },{\eta } ,1-2{\eta } ),\quad \text {with}\quad {\eta }= \frac{1}{3}\ell _2(x + \gamma -\gamma x), \\ \overline{CF}_{abcc}&=(1-2\delta , \delta ,\delta ),\quad \text {with}\quad \delta = \frac{1}{3}\ell _3(1-\gamma + \gamma x), \\ \overline{CF}_{aacc}&=(1-2\epsilon ,\epsilon , \epsilon ),\quad \text {with}\quad \epsilon = \frac{1}{3}\ell _1\ell _3(\gamma ^2h_1x +2\gamma (1-\gamma )+ (1-\gamma )^2h_2), \\ \overline{CF}_{bbcc}&=(1-2\zeta ,\zeta ,\zeta ),\quad \text {with}\quad \zeta = \frac{1}{3}x\ell _2\ell _3. \end{aligned}$$
- The ideal defining $\mathcal {V}_{N_{6-3_2}}\subset \mathbb {C}^{18}$ is ${\mathcal {I}}(N_{6-3_2})={\mathcal {J}}(N_{6-3_2})={\mathcal {J}}(T_6)$. The variety $\mathcal {V}_{N_{6-3_2}}$ has dimension 6.
- None of the numerical parameters $\gamma$, *x*, $\ell _1$, $\ell _2$, $\ell _3$, $h_1$, $h_2$ can be determined from quartet *CF*s. They can be determined with one degree of freedom, for instance if $\ell _3$ is given:
  $$\begin{aligned} \begin{aligned} \gamma&= \frac{(\ell _3-3CF_{ac|bc})(\ell _3CF_{ab|bc}-CF_{bc|bc})}{(\ell _3^2CF_{ab|bc}-2\ell _3CF_{bc|bc} + 3CF_{bc|bc}CF_{ac|bc})}, \quad x = \frac{CF_{bc|bc}(\ell _3-3CF_{ac|bc})}{\ell _3(\ell _3CF_{ab|bc}-CF_{bc|bc})}, \\ \ell _1&= \frac{-g}{(CF_{bc|bc}-3CF_{ab|bc}CF_{ac|bc})(\ell _3-3CF_{ac|bc})} ,\quad \ell _2 = \frac{3(\ell _3CF_{ab|bc}-CF_{bc|bc})}{(\ell _3-3CF_{ac|bc})}, \\ h_1&= \frac{g_{h_1}(CF_{bc|bc}-3CF_{ab|bc}CF_{ac|bc})}{g(\ell _3-3CF_{ac|bc})(\ell _3CF_{ab|bc}-CF_{bc|bc})},\quad h_2 = \frac{g_{h_2}(\ell _3-3CF_{ac|bc})}{g\ell _3(CF_{bc|bc}-3CF_{ab|bc}CF_{ac|bc})}. \\ \end{aligned} \end{aligned}$$
  where
  $$\begin{aligned} g&= \ell _3^2CF_{ab|ab} - 3\ell _3^2CF_{ab|ac}CF_{ab|bc} - 3\ell _3CF_{ab|ab}CF_{ac|bc} + 3\ell _3CF_{ac|ac}CF_{ab|bc} \\&\quad - 3CF_{bc|bc}CF_{ac|ac} + 9CF_{bc|bc}CF_{ab|ac}CF_{ac|bc}, \\ g_{h_1}&= \ell _3^3CF_{ab|ab} - 6\ell _3^3CF_{ab|ac}CF_{ab|bc} + 6\ell _3^2CF_{bc|bc}CF_{ab|ac} - 3\ell _3^2CF_{ab|ab}CF_{ac|bc} \\&\quad + 6\ell _3^2CF_{ac|ac}CF_{ab|bc} - 9\ell _3CF_{bc|bc}CF_{ac|ac} + 9CF_{bc|bc}CF_{ac|ac}CF_{ac|bc}, \\ g_{h_2}&= 2\ell _3^3CF_{ab|ab}CF_{ab|bc} - 6\ell _3^3CF_{ab|ac}CF_{ab|bc}^2 - 3\ell _3^2CF_{bc|bc}CF_{ab|ab} \\&\quad + 12\ell _3^2CF_{bc|bc}CF_{ab|ac}CF_{ab|bc} - 6\ell _3^2CF_{ab|ab}CF_{ab|bc}CF_{ac|bc} + 3\ell _3^2CF_{ac|ac}CF_{ab|bc}^2 \\&\quad - 6\ell _3CF_{bc|bc}^2CF_{ab|ac} + 12\ell _3CF_{bc|bc}CF_{ab|ab}CF_{ac|bc} - 6\ell _3CF_{bc|bc}CF_{ac|ac}CF_{ab|bc} \\&\quad + 3CF_{bc|bc}^2CF_{ac|ac} - 9CF_{bc|bc}CF_{ab|ab}CF_{ac|bc}^2. \end{aligned}$$

#### Proposition 37

Let the parameterized variety ${\mathcal {V}}_D$ be as in Proposition 12.

- ${\mathcal {V}}_D$ is defined by the 0 ideal, and thus ${\mathcal {V}}_D ={\mathbb {C}}^6$.
- From a point $(p_1,p_2,p_3,p_4,p_5,p_6)$ in the image of the parameterization of ${\mathcal {V}}_D$, none of the parameters can be determined. They can be determined with 1 degree of freedom, for instance, if $\ell _3$ is given:
  $$\begin{aligned} \gamma&= -\frac{3p_4\ell _3^2+(-3p_1p_4+p_3-3p_4-1)\ell _3+p_1p_3-p_1-p_3+1}{3p_4\ell _3^2+(2p_3-2)\ell _3+p_1p_3-p_1-p_3+1},\\ x&= -\frac{(p_3-1)(\ell _3+p_1-1)}{\ell _3(3p_4\ell _3+p_3-1)},\\ \ell _1&= \big [ (9p_4p_5+3p_6)\ell _3^2+(-3p_2p_4+3p_1p_6+3p_4-3p_6)\ell _3+3p_1p_3p_5-p_2p_3-3p_1p_5-3p_3p_5\\&\quad +p_2+p_3+3p_5-1 \big ]/{(3p_1p_4-p_3-3p_4+1)(\ell _3+p_1-1)},\\ \ell _2&= \frac{3p_4\ell _3-p_3+1}{\ell _3+p_1-1},\\ h_1&= \frac{f_1}{g_1}, \qquad h_2 = \frac{f_2}{g_2}, \end{aligned}$$
  where
  $$\begin{aligned} f_1&= -(54p_4^2p_5-18p_4p_6)\ell _3^4+(-54p_1p_4^2p_5+9p_2p_4^2+36p_3p_4p_5-54p_4^2p_5-36p_1p_4p_6\\&\quad -9p_4^2-36p_4p_5\\&\quad -9p_3p_6+36p_4p_6+9p_6)\ell _3^3 +(9p_1p_2p_4^2+36p_1p_3p_4p_5-18p_1^2p_4p_6+6p_2p_3p_4\\&\quad -9p_1p_4^2-9p_2p_4^2\\&\quad +6p_3^2p_5-36p_1p_4p_5-36p_3p_4p_5 -21p_1p_3p_6+36p_1p_4p_6-6p_2p_4-6p_3p_4\\&\quad +9p_4^2-12p_3p_5\\&\quad +36p_4p_5+21p_1p_6+21p_3p_6-18p_4p_6 +6p_4+6p_5-21p_6)\ell _3^2\\&\quad +(6p_1p_2p_3p_4+6p_1p_3^2p_5-15p_1^2p_3p_6p_2p_3^2-6p_1p_2p_4-6p_1p_3p_4-6p_2p_3p_4\\&\quad -12p_1p_3p_5-6p_3^2p_5\\&\quad +15p_1^2p_6+30p_1p_3p_6-2p_2p_3-p_3^2+6p_1p_4+6p_2p_4+6p_3p_4+6p_1p_5\\&\quad +12p_3p_5-30p_1p_6\\&\quad -15p_3p_6+p_2+2p_3-6p_4-6p_5+15p_6-1)\ell _3 -3p_1^3p_3p_6+p_1p_2p_3^2+3p_1^3p_6\\&\quad +9p_1^2p_3p_6\\&\quad -2p_1p_2p_3-p_1p_3^2-p_2p_3^2-9p_1^2p_6-9p_1p_3p_6 +p_1p_2+2p_1p_3+2p_2p_3\\&\quad +p_3^2+9p_1p_6+3p_3p_6\\&\quad -p_1-p_2-2p_3-3p_6+1,\\ g_1&= \ell _3(3p_1p_4-p_3-3p_4+1) \big ((9p_4p_5-3p_6)\ell _3^2+(3p_2p_4-3p_1p_6-3p_4+3p_6)\ell _3\\&\quad -3p_1p_3p_5+p_2p_3+3p_1p_5+3p_3p_5-p_2-p_3-3p_5+1\big ),\\ f_2&= +(54p_1p_4^2p_5+18p_3p_4p_5+54p_4^2p_5+9p_1p_4p_6-18p_4p_5-3p_3p_6-9p_4p_6+3p_6)\ell _3^3\\&\quad +(-18p_1p_2p_4^2-18p_1p_3p_4p_5+9p_1^2p_4p_6+6p_2p_3p_4+18p_1p_4^2+18p_2p_4^2+6p_3^2p_5 \\&\quad +18p_1p_4p_5+18p_3p_4p_5-3p_1p_3p_6-18p_1p_4p_6-6p_2p_4-6p_3p_4-18p_4^2-12p_3p_5\\&\quad -18p_4p_5+3p_1p_6+3p_3p_6+9p_4p_6+6p_4+6p_5-3p_6)\ell _3^2 \\&\quad +(-9p_1p_2p_3p_4+3p_2p_3^2+9p_1p_2p_4+9p_1p_3p_4+9p_2p_3p_4-6p_2p_3-3p_3^2-9p_1p_4\\&\quad -9p_2p_4-9p_3p_4+3p_2+6p_3+9p_4-3)\ell _3 \\&\quad -3p_1^2p_2p_3p_4+p_1p_2p_3^2+3p_1^2p_2p_4+3p_1^2p_3p_4+6p_1p_2p_3p_4-2p_1p_2p_3-p_1p_3^2-p_2p_3^2\\&\quad -3p_1^2p_4-6p_1p_2p_4-6p_1p_3p_4-3p_2p_3p_4+p_1p_2+2p_1p_3+2p_2p_3+p_3^2+6p_1p_4+3p_2p_4 \\&\quad +3p_3p_4-p_1-p_2-2p_3-3p_4+1,\\ g_2&= (\ell _3+p_1-1)(3p_4\ell _3+p_3-1)\cdot \big ((9p_4p_5-3p_6)\ell _3^2 +(3p_2p_4-3p_1p_6-3p_4+3p_6)\ell _3\\&\quad -3p_1p_3p_5+p_2p_3+3p_1p_5+3p_3p_5-p_2-p_3-3p_5+1\big ).\\ \end{aligned}$$

### B.3 Propositions from 4-Cycle Computations

#### Proposition 38

Consider the 5-taxon level-1 network $N_s$ with a single 4-cycle shown in Fig. 9 (left). Then

- The quartet concordance factors of $N_s$ are
  $$\begin{aligned} \overline{CF}_{abcd}&= \left( -\frac{1}{3}(2\gamma x_1 + \gamma x_2 - 3\gamma - x_2), \frac{1}{3}(\gamma x_1 - \gamma x_2 + x_2), 1 + \frac{1}{3}(\gamma x_1 + 2\gamma x_2 - 3\gamma - 2x_2) \right) ,\\ \overline{CF}_{xyzw}&=\left( 1-\frac{2}{3}\ell u_{xyzw}, \frac{1}{3} \ell u_{xyzw}, \frac{1}{3} \ell u_{xyzw} \right) \text { for } xyzw=aabc,\ aabd,\ aacd, \end{aligned}$$
  where
  $$\begin{aligned} u_{aabc}&= \gamma ^2h_2x_2 + \gamma ^2h_1 + \gamma ^2x_1 - \gamma h_2x_2 - 3\gamma ^2 - 2\gamma x_1 + 3\gamma + x_1, \\ u_{aabd}&= \gamma ^2x_1x_2 + \gamma ^2h_1 + \gamma ^2h_2 - 2\gamma x_1x_2 - 3\gamma ^2 - \gamma h_2 + x_1x_2 + 3\gamma , \\ u_{aacd}&= \gamma ^2h_1x_1 + \gamma ^2h_2 + \gamma ^2x_2 - 3\gamma ^2 - \gamma h_2 - 2\gamma x_2 + 3\gamma + x_2. \end{aligned}$$
- The ideal defining $\mathcal {V}_{N_s}\subset \mathbb {C}^{12}$ is ${\mathcal {I}}(N_s) ={\mathcal {J}}(N_s)$. The variety ${\mathcal {V}}_{N_s}$ has dimension 5.
- None of the numerical parameters $x_1$, $x_2$, $h_1$, $h_2$, $\ell$, $\gamma$ can be determined from quartet *CF*s. They can be determined with 1 degree of freedom, for instance, if $\gamma$ is given:
  $$\begin{aligned} \begin{aligned} x_1&= \frac{\gamma -CF_{ab|cd} + CF_{ac|bd}}{\gamma }, \quad x_2 = \frac{\gamma - CF_{ab|cd} - 2CF_{ac|bd}}{\gamma - 1}, \\ h_1&= \frac{n_{h_1}}{d_h}, \quad h_2 = \frac{n_{h_2}}{d_h}, \quad \ell = \frac{n_{\ell }}{d_{\ell }}, \end{aligned} \end{aligned}$$
  where
  $$\begin{aligned} n_{h_1}&= \Big ((CF_{ab|ac} - 2CF_{ab|ad})CF_{ab|cd} + (CF_{ab|cd} - 1)CF_{ac|ad} - (CF_{ab|ac} + CF_{ab|ad}\\&\quad - 2cf_{ac|ad})CF_{ac|bd} + CF_{ab|ad}\Big )\gamma ^3 - \Big ((CF_{ab|ac} + CF_{ab|ad})CF_{ab|cd}^2 - 2(CF_{ab|ac}\\&\quad - 2CF_{ab|ad})CF_{ac|bd}^2 + 2(CF_{ab|ac} - 2CF_{ab|ad})CF_{ab|cd} - 3(CF_{ab|cd}^2 - CF_{ab|cd})CF_{ac|ad}\\&\quad + ((CF_{ab|ac} + 4CF_{ab|ad})CF_{ab|cd} - 6CF_{ab|cd}CF_{ac|ad} - 2CF_{ab|ac} + CF_{ab|ad})CF_{ac|bd}\Big )\gamma ^2\\&\quad + \Big (4CF_{ac|ad}CF_{ac|bd}^3 + (2CF_{ab|ac} + CF_{ab|ad})CF_{ab|cd}^2 - 4(CF_{ab|ac} - CF_{ab|ad})CF_{ac|bd}^2\\&\quad + (CF_{ab|ac} - 3CF_{ab|ad})CF_{ab|cd} - (CF_{ab|cd}^3 + 3CF_{ab|cd}^2 - 3CF_{ab|cd} - 1)CF_{ac|ad}\\&\quad + (2(CF_{ab|ac} + 2CF_{ab|ad})CF_{ab|cd} - 3(CF_{ab|cd}^2 + 2CF_{ab|cd} + 1)CF_{ac|ad} - CF_{ab|ac}\\&\quad + 3CF_{ab|ad})CF_{ac|bd} - CF_{ab|ad}\Big )\gamma -4CF_{ac|ad}CF_{ac|bd}^3 - CF_{ab|ac}CF_{ab|cd}^2 + 2CF_{ab|ac}CF_{ac|bd}^2\\&\quad + CF_{ab|ad}CF_{ab|cd} + (CF_{ab|cd}^3 - CF_{ab|cd})CF_{ac|ad} - (CF_{ab|ac}CF_{ab|cd} - (3CF_{ab|cd}^2 + 1)CF_{ac|ad}\\&\quad + CF_{ab|ad})CF_{ac|bd}, \\ n_{h_2}&= \Big ((CF_{ab|ac} - 2CF_{ab|ad})CF_{ab|cd} + (CF_{ab|cd} - 1)CF_{ac|ad} - (CF_{ab|ac} + CF_{ab|ad}\\&\quad - 2CF_{ac|ad})CF_{ac|bd} + CF_{ab|ad}\Big )\gamma ^3 + \Big ((3CF_{ab|ac} - CF_{ab|ad})CF_{ab|cd}^2 - (3CF_{ab|ac} + CF_{ab|ad}\\&\quad - 2CF_{ac|ad})CF_{ac|bd}^2 - CF_{ab|ac}CF_{ab|cd} - (CF_{ab|cd}^2 - 1)CF_{ac|ad} + (2CF_{ab|ad}CF_{ab|cd}\\&\quad - (CF_{ab|cd} + 3)CF_{ac|ad} + CF_{ab|ac} + 3CF_{ab|ad})CF_{ac|bd} - CF_{ab|ad}\Big )\gamma ^2- \Big (CF_{ab|ac}CF_{ab|cd}^3\\&\quad + 2CF_{ab|ac}CF_{ac|bd}^3 + (3CF_{ab|ac} - 2CF_{ab|ad})CF_{ab|cd}^2- (3CF_{ab|ac}CF_{ab|cd} + 3CF_{ab|ac}\\&\quad + 2CF_{ab|ad} - 2CF_{ac|ad})CF_{ac|bd}^2 - 2CF_{ab|ad}CF_{ab|cd} - (CF_{ab|cd}^2 - CF_{ab|cd})CF_{ac|ad}\\&\quad + (4CF_{ab|ad}CF_{ab|cd} - (CF_{ab|cd} + 1)CF_{ac|ad} + 2CF_{ab|ad})CF_{ac|bd}\Big )\gamma + CF_{ab|ac}CF_{ab|cd}^3\\&\quad + 2CF_{ab|ac}CF_{ac|bd}^3 - CF_{ab|ad}CF_{ab|cd}^2 + 2CF_{ab|ad}CF_{ab|cd}CF_{ac|bd} - (3CF_{ab|ac}CF_{ab|cd}\\&\quad + CF_{ab|ad})CF_{ac|bd}^2, \\ d_h&= \Big ((CF_{ab|ac} - 2CF_{ab|ad})CF_{ab|cd} + (CF_{ab|cd} - 1)CF_{ac|ad} - (CF_{ab|ac} + CF_{ab|ad}\\&\quad - 2CF_{ac|ad})CF_{ac|bd} + CF_{ab|ad}\Big )\gamma ^3+ \Big (CF_{ab|ad}CF_{ab|cd}^2 - 2CF_{ab|ad}CF_{ac|bd}^2\\&\quad - CF_{ab|ac}CF_{ab|cd} + (CF_{ab|ad}CF_{ab|cd} + CF_{ab|ac})CF_{ac|bd}\Big )\gamma ^2, \\ n_{\ell }&= + 3\Big ((CF_{ab|ac} - 2CF_{ab|ad})CF_{ab|cd} + (CF_{ab|cd} - 1)CF_{ac|ad} - (CF_{ab|ac} + CF_{ab|ad}\\&\quad - 2CF_{ac|ad})CF_{ac|bd}+ CF_{ab|ad}\Big )\gamma ^2+ 3\Big (CF_{ab|ad}CF_{ab|cd}^2 - 2CF_{ab|ad}CF_{ac|bd}^2\\&\quad - CF_{ab|ac}CF_{ab|cd} + (CF_{ab|ad}CF_{ab|cd} + CF_{ab|ac})CF_{ac|bd}\Big )\gamma ,\\ d_{\ell }&= -\Big (4CF_{ab|cd}^3 - 6CF_{ab|cd}CF_{ac|bd}^2 - 4CF_{ac|bd}^3 - 3CF_{ab|cd}^2\\&\quad + (6CF_{ab|cd}^2 + 3CF_{ab|cd} + 1)CF_{ac|bd} - CF_{ab|cd}\Big )\gamma ^2 \\&\quad + \Big (CF_{ab|cd}^4 - 4(CF_{ab|cd} + 1)CF_{ac|bd}^3 + 4CF_{ac|bd}^4 + 4CF_{ab|cd}^3\\&\quad - (3CF_{ab|cd}^2 + 6CF_{ab|cd} + 1)CF_{ac|bd}^2 - 4CF_{ab|cd}^2 \\&\quad + (2CF_{ab|cd}^3 + 6CF_{ab|cd}^2 + 5CF_{ab|cd} + 1)CF_{ac|bd} - CF_{ab|cd}\Big )\gamma -CF_{ab|cd}^4 + 4CF_{ab|cd}CF_{ac|bd}^3 \\&\quad - 4CF_{ac|bd}^4+ (3CF_{ab|cd}^2 + 1)CF_{ac|bd}^2 + CF_{ab|cd}^2 - 2(CF_{ab|cd}^3 + CF_{ab|cd})CF_{ac|bd}. \end{aligned}$$

#### Proposition 39

Let $N_w$ be the 5-taxon level-1 network with a single 4-cycle shown in Fig. 9 (center). Then

- The quartet concordance factors of $N_w$ are
  $$\begin{aligned} \overline{ CF}_{abcd}&= \left( \frac{1}{3}(-2\gamma x_1-\gamma x_2 + 3\gamma + x_2), \frac{1}{3}(\gamma x_1-\gamma x_2 + x_2), 1 + \frac{1}{3} (\gamma x_1 + 2\gamma x_2-3\gamma -2x_2) \right) ,\\ \overline{CF}_{xyzw}&= \left( 1 - \frac{2}{3}\ell u_{xyzw}, \frac{1}{3}\ell u_{xyzw}, \frac{1}{3}\ell u_{xyzw} \right) \text { for } xyzw=aabc,\ aabd,\ aacd, \end{aligned}$$
  where
  $$\begin{aligned} u_{aabc}&=x_1,\\ u_{aabd}&= -\gamma x_1 + \gamma + x_1,\\ u_{aacd}&=-\gamma x_1x_2 + x_1x_2 + \gamma . \end{aligned}$$
- The ideal defining $\mathcal {V}_{N_w}\subset \mathbb {C}^{12}$ is
  $$\begin{aligned} {\mathcal {I}}(N_w) = {\mathcal {J}}(N_w) + \langle CF_{ab|cd}CF_{ab|ac} - CF_{ac|bd}CF_{ab|ac} - CF_{ab|ad} + CF_{ac|ad} \rangle , \end{aligned}$$
  and $\mathcal {V}_{N_w}$ has dimension 4.
- The numerical parameters $x_1$, $x_2$, $\ell$, $\gamma$ can be determined from quartet *CF*s:
  $$\begin{aligned} x_1&= \frac{3CF_{ab|ac}CF_{ac|bd} - CF_{ab|ac} + CF_{ab|ad} - CF_{ac|ad}}{CF_{ab|ac} - CF_{ab|ad}} ,\\ x_2&=- \big [ CF_{ab|ad}CF_{ab|cd} - (CF_{ab|cd} - 2)CF_{ac|ad} - (CF_{ab|ad} + 2CF_{ac|ad})CF_{ac|bd} + CF_{ab|ac}\\&\quad - 2CF_{ab|ad} \big ]/{CF_{ab|ad}CF_{ab|cd} + 2CF_{ab|ad}CF_{ac|bd} - CF_{ab|ac}}, \\ \ell&= \frac{3(CF_{ab|ac} - CF_{ab|ad})}{CF_{ab|cd} + 2CF_{ac|bd} - 1}, \\ \gamma&= -\frac{CF_{ab|ad}CF_{ab|cd} - (3CF_{ab|ac} - 2CF_{ab|ad})CF_{ac|bd} + CF_{ab|ac} - 2CF_{ab|ad} + CF_{ac|ad}}{3CF_{ab|ac}CF_{ac|bd} - 2CF_{ab|ac} + 2CF_{ab|ad} - CF_{ac|ad}}. \end{aligned}$$

#### Proposition 40

Let $N_n$ be the 5-taxon level-1 network with a single 4-cycle shown in Fig. 9 (right). Then

- The quartet concordance factors of $N_n$ are
  $$\begin{aligned} CF_{abcd}&= \left( \frac{1}{3}(-2\gamma x_1-\gamma x_2 + 3\gamma + x_2), \frac{1}{3}(\gamma x_1-\gamma x_2 + x_2), 1 + \frac{1}{3}(\gamma x_1 + 2\gamma x_2-3\gamma -2x_2) \right) ,\\ CF_{xyzw}&= \left( 1 - \frac{2}{3}\ell u_{xyzw}, \frac{1}{3}\ell u_{xyzw}, \frac{1}{3}\ell u_{xyzw} \right) \text { for } xyzw=aabc,\ aabd,\ aacd, \end{aligned}$$
  where
  $$\begin{aligned} u_{aabc} =\gamma + x_2 - \gamma x_2, \quad u_{aabd}=1,\quad u_{aacd} = 1 - \gamma + \gamma x_1. \end{aligned}$$
- the ideal defining $\mathcal {V}_{N_n}\subset \mathbb {C}^{12}$ is
  $$\begin{aligned} {\mathcal {I}}(N_n)&= {\mathcal {J}}(N_n) + \langle 3CF_{ac|bd}CF_{ad|ab} + CF_{ad|ab} - CF_{ac|ab} - CF_{ac|ad}, \\&\qquad CF_{ab|cd}CF_{ac|ab} + CF_{ab|cd}CF_{ac|ad} - CF_{ac|bd}CF_{ac|ab} + 2CF_{ac|bd}CF_{ac|ad} - CF_{ac|ab}, \\&\qquad 3CF_{ab|cd}CF_{ad|ab} - 2CF_{ad|ab} - CF_{ac|ab} + 2CF_{ac|ad} \rangle , \end{aligned}$$
  and $\mathcal {V}_{N_n}$ has dimension 3.
- The parameter $\ell$ can be identified from quartet *CF*s:
  $$\begin{aligned} \ell = 3CF_{a_1ba_2d}, \end{aligned}$$
  but none of $x_1$, $x_2$, and $\gamma$ can be. However, the quantities $x_1\gamma - \gamma$ and $x_2(\gamma -1)-\gamma$ can be determined from the quartet *CF*s, so if $\gamma$ is known:
  $$\begin{aligned} x_1 = \frac{\gamma -CF_{abcd} + CF_{acbd}}{\gamma }, \quad x_2 = \frac{\gamma - CF_{abcd} - 2CF_{acbd}}{\gamma - 1}. \end{aligned}$$

#### Proposition 41

Let $N_{sn}$ be a 6-taxon level-1 network with a central 4-cycle as shown in Fig. 14 (right). Then

- The quartet concordance factors of $N_{sn}$ are
  $$\begin{aligned} \overline{CF}_{aabc}&=(1-2u,u,u) ,\quad \text {with}\quad u=\ell _1\left[ \frac{1}{3} \gamma ^2h_1+\frac{1}{3} (1-\gamma )^2 h_2x_2+ \gamma (1-\gamma )\left( 1-\frac{1}{3} x_1\right) \right] ,\\ \overline{CF}_{aabd}&=(1-2u,u,u) ,\quad \text {with}\quad u=\ell _1\left[ \frac{1}{3} \gamma ^2h_1+\frac{1}{3} (1-\gamma )^2 h_2+ \gamma (1-\gamma )\left( 1-\frac{1}{3} x_1x_2\right) \right] , \\ \overline{CF}_{aacc}&=(1-2u,u,u) ,\quad \text {with}\quad u= \ell _1\ell _2\left[ \frac{1}{3} \gamma ^2h_1x_1+\frac{1}{3} (1-\gamma )^2 h_2x_2+ \frac{2}{3}\gamma (1-\gamma ) \right] , \\ \overline{CF}_{aacd}&= (1-2u,u,u) ,\quad \text {with}\quad u= \ell _1\left[ \frac{1}{3} \gamma ^2h_1x_1+\frac{1}{3} (1-\gamma )^2 h_2+ \gamma (1-\gamma )\left( 1-\frac{1}{3} x_2\right) \right] , \\ \overline{CF}_{abcc}&= (u,u,1-2u), \quad \text {with}\quad u= \ell _2\frac{1}{3} \left( \gamma x_1 +(1-\gamma ) \right) , \\ \overline{CF}_{abcd}&= \left( \gamma \left( 1-\frac{2}{3} x_1 \right) +(1-\gamma )\frac{1}{3} x_2, \frac{1}{3} (\gamma x_1+(1-\gamma )x_2),\gamma \frac{1}{3} x_1+(1-\gamma )\left( 1-\frac{2}{3} x_2\right) \right) , \\ \overline{CF}_{accd}&= (u,u,1-2u), \quad \text {with}\quad u=\frac{1}{3}\ell _2(\gamma +(1-\gamma )x_2), \\ \overline{CF}_{bccd}&= (u,u,1-2u), \quad \text {with}\quad u=\frac{1}{3}\ell _2. \end{aligned}$$
- The ideal defining $\mathcal {V}({N_{sn}})\subset \mathbb {C}^{9}$ is
  $$\begin{aligned} {\mathcal {I}}(N_{sn})&= {\mathcal {J}}(N_{sn}) + \langle C_{ab|cd}C_{ac|cd+}C_{ab|cd}C_{ac|bc}-C_{ac|cd}C_{ac|bd}-C_{ac|cd}\\&\quad +2C_{ac|bd}C_{ac|bc}, \quad 3C_{bc|cd}C_{ac|bd}+C_{bc|cd}-C_{ac|cd}-C_{ac|bc},\\&\quad 3C_{bc|cd}C_{ab|cd}-2C_{bc|cd}-C_{ac|cd}+2C_{ac|bc} \rangle . \end{aligned}$$
  The variety $\mathcal {V}_{N_{sn}}$ has dimension 7.
- The numerical parameters $\gamma$, $h_1$, $h_2$, $x_1$, $x_2$, $\ell _1$, $\ell _2$ can be determined from quartet *CF*s: small
  $$\begin{aligned} \begin{aligned} \gamma&= \frac{n_{\gamma }}{d_{\gamma }}, \quad h_1 = \frac{n_{h_1}}{d_{h_1}}, \quad h_2 = \frac{n_{h_2}}{d_{h_2}}, \quad x_1 = \frac{n_{x_1}}{d_{x_1}}, \quad x_2 = \frac{n_{x_2}}{d_{x_2}},\\ \ell _1&= \frac{-3C_{ab|ac}C_{bc|cd}+3C_{ab|ad}C_{bc|cd}-3C_{bc|cd}C_{ac|ad} +C_{ac|ac}}{-C_{ab|cd}C_{ac|bc}-2CacbdC_{ac|bc}-C_{bc|cd}+C_{ac|cd}+C_{ac|bc}}, \quad \ell _2 = 3C_{bc|cd}. \end{aligned} \end{aligned}$$
  where
  $$\begin{aligned} n_{\gamma }&=-C_{ac|ac}C_{ab|cd}^2-C_{ac|ac}C_{ab|cd}Cacbd+2C_{ac|ac}Cacbd^2-3C_{ab|ac}C_{ab|cd}C_{ac|bc}\\&\quad -3C_{ab|cd}C_{ac|ad}C_{ac|bc}-6C_{ab|ac}CacbdC_{ac|bc}-6C_{ac|ad}CacbdC_{ac|bc}-3C_{ab|ad}C_{bc|cd}\\&\quad -C_{ac|ac}C_{ab|cd}+3C_{bc|cd}C_{ac|ad}+3C_{ab|ac}C_{ac|cd}+3C_{ac|ad}C_{ac|cd}+C_{ac|ac}Cacbd\\&\quad +3C_{ab|ad}C_{ac|bc}-3C_{ac|ad}C_{ac|bc},\\ d_{\gamma }&= -3C_{ab|ac}C_{bc|cd}-4C_{ac|ac}C_{ab|cd}+3C_{bc|cd}C_{ac|ad}+6C_{ab|ac}C_{ac|cd}-3C_{ab|ad}C_{ac|cd}\\&\quad +3C_{ac|ad}C_{ac|cd}-2C_{ac|ac}Cacbd-3C_{ab|ac}C_{ac|bc}+3C_{ab|ad}C_{ac|bc}-6C_{ac|ad}C_{ac|bc}+2C_{ac|ac},\\ n_{h_1}&= (-3C_{ac|ad}C_{ac|cd}+C_{ac|ac})x_1+6C_{ab|ac}C_{bc|cd}-3C_{ab|ad}C_{bc|cd}+3C_{ac|ac}C_{ab|cd}\\&\quad -6C_{ab|ac}C_{ac|cd}+3C_{ab|ad}C_{ac|cd}+3C_{ac|ac}Cacbd+3C_{ac|ad}C_{ac|bc}-3C_{ac|ac},\\ d_{h_1}&= +3C_{ab|ac}C_{bc|cd}-3C_{ab|ad}C_{bc|cd}-C_{ac|ac}C_{ab|cd}+3C_{bc|cd}C_{ac|ad}+C_{ac|ac}Cacbd\\&\quad -3C_{ab|ac}C_{ac|bc}+3C_{ab|ad}C_{ac|bc}-3C_{ac|ad}C_{ac|bc},\\ n_{h_2}&= (3C_{ab|ac}C_{ac|bc}-C_{ac|ac})x_2+3C_{ab|ad}C_{bc|cd}+3C_{ac|ac}C_{ab|cd}-6C_{bc|cd}C_{ac|ad}\\&\quad -3C_{ab|ac}C_{ac|cd}-3C_{ab|ad}C_{ac|bc}+6C_{ac|ad}C_{ac|bc},\\ d_{h_2}&= -3C_{ab|ac}C_{bc|cd}+3C_{ab|ad}C_{bc|cd}-C_{ac|ac}C_{ab|cd}-3C_{bc|cd}C_{ac|ad}+3C_{ab|ac}C_{ac|cd}\\&\quad -3C_{ab|ad}C_{ac|cd}+3C_{ac|ad}C_{ac|cd}-2C_{ac|ac}Cacbd+C_{ac|ac},\\ n_{x_1}&= +3C_{ab|ac}C_{bc|cd}-3C_{ab|ad}C_{bc|cd}+3C_{ac|ac}C_{ab|cd}-3C_{ab|ac}C_{ac|cd}\\&\quad +3C_{ab|ad}C_{ac|cd}+3C_{ab|ac}C_{ac|bc}-3C_{ab|ad}C_{ac|bc}+6C_{ac|ad}C_{ac|bc}-3C_{ac|ac},\\ d_{x_1}&= -3C_{ab|ad}C_{bc|cd}-C_{ac|ac}C_{ab|cd}+3C_{bc|cd}C_{ac|ad}+3C_{ab|ac}C_{ac|cd}\\&\quad +3C_{ac|ad}C_{ac|cd}-2C_{ac|ac}Cacbd-C_{ac|ac},\\ n_{x_2}&= +3C_{ab|ad}C_{bc|cd}+3C_{ac|ac}C_{ab|cd}-3C_{bc|cd}C_{ac|ad}-6C_{ab|ac}C_{ac|cd}\\&\quad +3C_{ab|ad}C_{ac|cd}-3C_{ac|ad}C_{ac|cd}+3C_{ac|ac}Cacbd-3C_{ab|ad}C_{ac|bc}+3C_{ac|ad}C_{ac|bc},\\ d_{x_2}&= -3C_{ab|ac}C_{bc|cd}+3C_{ab|ad}C_{bc|cd}-C_{ac|ac}C_{ab|cd}+C_{ac|ac}Cacbd\\&\quad -3C_{ab|ac}C_{ac|bc}-3C_{ac|ad}C_{ac|bc}+2C_{ac|ac}.\\ \end{aligned}$$
